# Development and Validation of a Preoperative Magnetic Resonance Imaging Radiomics–Based Signature to Predict Axillary Lymph Node Metastasis and Disease-Free Survival in Patients With Early-Stage Breast Cancer

**DOI:** 10.1001/jamanetworkopen.2020.28086

**Published:** 2020-12-08

**Authors:** Yunfang Yu, Yujie Tan, Chuanmiao Xie, Qiugen Hu, Jie Ouyang, Yongjian Chen, Yang Gu, Anlin Li, Nian Lu, Zifan He, Yaping Yang, Kai Chen, Jiafan Ma, Chenchen Li, Mudi Ma, Xiaohong Li, Rong Zhang, Haitao Zhong, Qiyun Ou, Yiwen Zhang, Yufang He, Gang Li, Zhuo Wu, Fengxi Su, Erwei Song, Herui Yao

**Affiliations:** 1 Guangdong Provincial Key Laboratory of Malignant Tumor Epigenetics and Gene Regulation, Breast Tumor Centre, Department of Medical Oncology, Phase I Clinical Trial Centre, Sun Yat-sen Memorial Hospital, Sun Yat-sen University, Guangzhou, China; 2 Department of Radiology, Shunde Hospital, Southern Medical University, Foshan, China; 3 Department of Breast Surgery, Tungwah Hospital, Sun Yat-sen University, Dongguan, China; 4 Department of Medical Oncology, The Third Affiliated Hospital of Sun Yat-sen University, Guangzhou, China; 5 Guangdong Medical University, Zhanjiang, China; 6 Imaging Diagnostic and Interventional Center, Sun Yat-sen University Cancer Center, State Key Laboratory of Oncology in South China, Collaborative Innovation Center for Cancer Medicine, Guangzhou, China; 7 Guangzhou Regenerative Medicine and Health Guangdong Laboratory, Fountain-Valley Institute for Life Sciences, Guangzhou Institute of Biomedicine and Health, Chinese Academy of Sciences, Huangpu District, Guangzhou, China

## Abstract

**Importance:**

Axillary lymph node metastasis (ALNM) status, typically estimated using an invasive procedure with a high false-negative rate, strongly affects the prognosis of recurrence in breast cancer. However, preoperative noninvasive tools to accurately predict ALNM status and disease-free survival (DFS) are lacking.

**Objective:**

To develop and validate dynamic contrast–enhanced magnetic resonance imaging (DCE-MRI) radiomic signatures for preoperative identification of ALNM and to assess individual DFS in patients with early-stage breast cancer.

**Design, Setting, and Participants:**

This retrospective prognostic study included patients with histologically confirmed early-stage breast cancer diagnosed at 4 hospitals in China from July 3, 2007, to September 21, 2019, randomly divided (7:3) into development and vaidation cohorts. All patients underwent preoperative MRI scans, were treated with surgery and sentinel lymph node biopsy or ALN dissection, and were pathologically examined to determine the ALNM status. Data analysis was conducted from February 15, 2019, to March 20, 2020.

**Exposure:**

Clinical and DCE-MRI radiomic signatures.

**Main Outcomes and Measures:**

The primary end points were ALNM and DFS.

**Results:**

This study included 1214 women (median [IQR] age, 47 [42-55] years), split into development (849 [69.9%]) and validation (365 [30.1%]) cohorts. The radiomic signature identified ALNM in the development and validation cohorts with areas under the curve (AUCs) of 0.88 and 0.85, respectively, and the clinical-radiomic nomogram accurately predicted ALNM in the development and validation cohorts (AUC, 0.92 and 0.90, respectively) based on a least absolute shrinkage and selection operator (LASSO)–logistic regression model. The radiomic signature predicted 3-year DFS in the development and validation cohorts (AUC, 0.81 and 0.73, respectively), and the clinical-radiomic nomogram could discriminate high-risk from low-risk patients in the development cohort (hazard ratio [HR], 0.04; 95% CI, 0.01-0.11; *P* < .001) and the validation cohort (HR, 0.04; 95% CI, 0.004-0.32; *P* < .001) based on a random forest–Cox regression model. The clinical-radiomic nomogram was associated with 3-year DFS in the development and validation cohorts (AUC, 0.89 and 0.90, respectively). The decision curve analysis demonstrated that the clinical-radiomic nomogram displayed better clinical predictive usefulness than the clinical or radiomic signature alone.

**Conclusions and Relevance:**

This study described the application of MRI-based machine learning in patients with breast cancer, presenting novel individualized clinical decision nomograms that could be used to predict ALNM status and DFS. The clinical-radiomic nomograms were useful in clinical decision-making associated with personalized selection of surgical interventions and therapeutic regimens for patients with early-stage breast cancer.

## Key Points

**Question:**

Can multiparametric magnetic resonance imaging (MRI) radiomic profiles be used to predict axillary lymph node metastasis (ALNM) and disease-free survival (DFS) in patients with early-stage breast cancer?

**Findings:**

In this prognostic study that included 1214 patients, 2 clinical-radiomic nomograms were developed that accurately predicted ALNM and stratified patients into low-risk and high-risk groups for DFS.

**Meaning:**

In this study, clinical-radiomic nomograms were useful in clinical decision-making associated with personalized selection of surgical interventions and therapeutic regimens for patients with early-stage breast cancer.

## Teaser

This prognostic study develops and validates dynamic contrast–enhanced magnetic resonance imaging (DCE-MRI) radiomic signatures for preoperative identification of axillary lymph node metastasis and to assess individual disease-free survival in patients with early-stage breast cancer.

## Introduction

Sentinel lymph node biopsy is the current standard approach in axillary lymph node (ALN) staging, which guides an oncologist’s decision for the implementation of ALN dissection (ALND), surgery, and subsequent treatment for patients with breast cancer. However, it is an invasive procedure and has been criticized because of its high false-negative rate. Moreover, it has low efficiency owing to the unavoidable extension of the surgery period because of the long wait time for the frozen section. It also has an uncertain diagnostic utility for neoadjuvant strategies.^1,2,3^ Axillary lymph node metastasis (ALNM) strongly affects the prognosis of breast cancer recurrence. Therefore, accurately evaluating ALNM status could optimize the treatment strategy and reduce the risk of recurrence.

Although several multigene-based expression assays, such as the 70-gene expression profile and the 21-gene recurrence score assay, have been developed to select patients who are most likely to develop metastatic relapse and to predict the benefit of postoperative chemotherapy,^4,5,6^ they are costly, and their utility has only been established in certain subsets of patients.^4,7,8,9^ Therefore, a precise and efficient diagnostic approach with a higher clinical applicability and generalizability is urgently needed to preoperatively estimate disease-free survival (DFS).

The advent of artificial intelligence in radiomics has emerged as a noninvasive and less costly approach in precision medicine for breast cancer.^10,11^ Radiomic high-dimensional features extracted from preoperative dynamic contrast–enhanced magnetic resonance imaging (DCE-MRI) offer an insight into the micrometastases of ALN that are imperceptible to human eyes, and they can expose aspects of intratumor heterogeneity that have potential prognostic relevance. In this study, we used what is, to our knowledge, the largest sample size to date to develop and validate DCE-MRI radiomic signatures for preoperative identification of ALNM and assessment of individual DFS in patients with early-stage breast cancer.

## Methods

### Study Design and Patients

In this multicenter, retrospective prognostic study, the preoperative DCE-MRIs and clinical data of 1717 patients with early-stage breast cancer were collected from 4 hospitals in China, of whom 1214 patients passed quality control for the final analysis, according to the Transparent Reporting of a Multivariable Prediction Model for Individual Prognosis or Diagnosis (TRIPOD) reporting guideline.^12^ A total of 622 patients were recruited from the Sun Yat-sen Memorial Hospital (SYSMH cohort) of Sun Yat-sen University, a national hospital (Guangzhou, China). The patients consisted of 128 patients from a prospective phase 3 trial (NCT01503905)^13^ and 494 patients from an independent retrospective cohort. A total of 381 patients were recruited from the Sun Yat-sen University Cancer Center (SYSUCC cohort), a national hospital (Guangzhou, China); 121 patients from the Shunde Hospital of Southern Medical University (SMUSH cohort) (Foshan, China); and 90 patients from the Tungwah Hospital of Sun Yat-sen University (SYSUTH cohort) (Dongguan, China). All patients were reassessed by 5 of us (Y. Yu, Y.T., Q.H., J.O., and H.Y.). The data were censored on February 4, 2020. The follow-up was performed according to the recommendation of the National Comprehensive Cancer Network guidelines.^1^ eTable 1 in the Supplement shows information from the 4 institutions. To build and validate the radiomic signature, patients from the 4 centers were divided randomly (7:3) into the development (n = 849 [69.9%]) and validation (n = 365 [30.1%]) cohorts. This study was conducted from February 15, 2019, to March 20, 2020. The study protocol was approved by the ethics committee of each participating hospital and was conducted in accordance with the Declaration of Helsinki^14^ and Good Clinical Practice guidelines. The requirement for informed consent from patients whose information was retrospectively collected from participant hospitals was waived. More details regarding the study flowchart, the inclusion and exclusion criteria, and the outcomes are described in eFigure 1 and the eAppendix in the Supplement.

### Radiomic Feature Extraction

**Figure 1.**
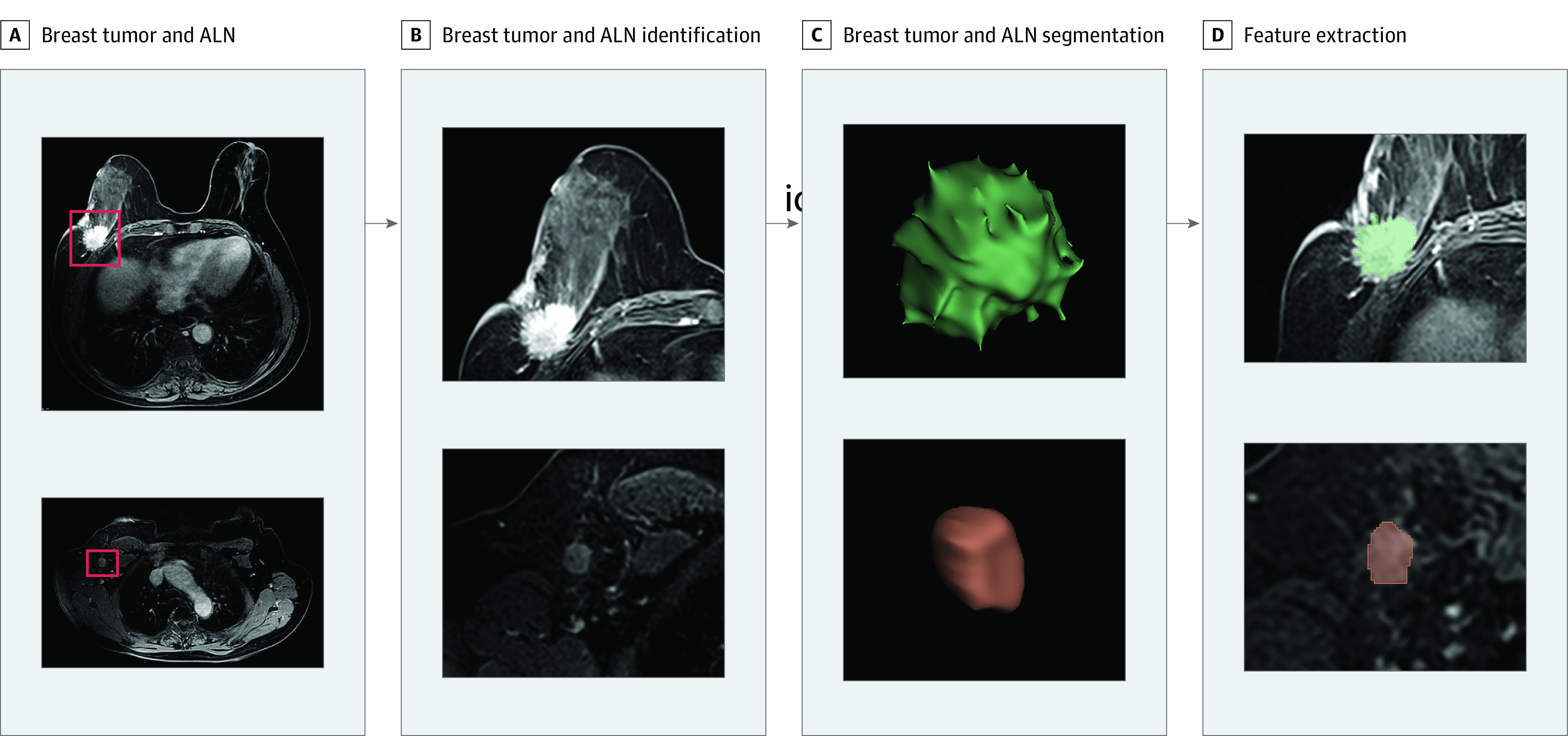
Workflow of Radiomic Feature Extraction ALN indicates axillary lymph node.

The radiomic workflow is shown in Figure 1, and the details of the MRI protocol are shown in eTable 2 in the Supplement. All DCE-MRIs were normalized to obtain a standard normal distribution of image intensities, using the N4ITK bias correction code. In addition, 3-dimensional regions of interest (ROIs) in the ALNs and tumors were separately semi-automatically segmented and delineated by multiple radiologists (Q.H., N.L., X.L., R.Z., Z.W., and C.X.), who were masked to the patients’ clinical outcomes using 3D Slicer^15,16^ version 4.10.2. After the ROIs of the ALNs and tumors were reconstructed and segmented, the volume of interest (VOI) images (Digital Imaging and Communications in Medicine format) were transferred to the SlicerRadiomics code. Then, an in-house texture extraction platform was developed based on the Python package PyRadiomics. Each VOI contrast-enhanced T1-weighted imaging (T1+C), T2-weighted imaging (T2WI), and diffusion-weighted imaging–quantitatively measured apparent diffusion coefficient (DWI-ADC) imaging had 6 groups of radiomic features. The voxel-based features included shape, first-order, gray-level cooccurrence matrix, gray-level size zone matrix, gray-level dependence matrix, and neighboring gray tone difference matrix, containing a total of 863 quantitative features. The ROIs were evaluated by radiologists from the same hospital following the Breast, Imaging, Reporting and Data System standards.^17^ This was used to define the boundary of the ROIs. Three of us (Q.H., Z.W., and C.X.), each with at least 15 years of experience in breast MRI, were chiefly responsible for the ROI evaluations, and any disagreements were resolved by consensus.

### Development of the Radiomic Signature

Unsupervised clustering^18^ of radiomic features and heatmaps were used to reveal patient clusters with similar radiomic expression patterns and their associations with the ALNM and DFS. Least absolute shrinkage and selector operation (LASSO)^19^ and random forest algorithm (the top 30 candidate)^20^ were used to feature high-dimensional imaging data from each of the ALN and tumor T1+C, T2WI, and DWI-ADC images. The key predictive combination of features was used to build the T1+C, T2WI, and DWI-ADC imaging signatures via linear combination or supporting vector machine (SVM) models. The ALN, tumor, or combination of tumor and ALN radiomic signatures were combined with the T1+C, T2WI, and DWI-ADC imaging signatures obtained using the logistic regression or SVM model to predict ALNM and with the penalized Cox regression to predict DFS. To find the most suitable base model for ALNM or DFS prediction tasks, the performances of the LASSO–logistic regression, LASSO-SVM, random forest–logistic regression, and random forest–SVM models in predicting ALNM status were compared; the performances of LASSO–Cox regression and random forest–Cox regression models in predicting DFS were also compared.

### Development of the Clinical-Radiomic Nomogram

A univariate analysis was used to assess the association between the clinical characteristics and ALNM or DFS, and then the significant clinical risk factors were used to develop and validate clinical signatures for ALNM and DFS prediction. A multivariable regression was applied to select independent predictors of ALNM and DFS from the radiomic and clinical signatures. Finally, by considering the clinical characteristics and radiomic signature covariates, a clinically applicable clinical-radiomic nomogram was developed that could predict ALNM and individual DFS.

### Statistical Analysis

The continuous variables were compared using the independent *t* test, and the 2-group categorical variables were compared using the χ^2^ test. DFS and overall survival (OS) were calculated using the Kaplan-Meier method and the log-rank test, and the hazard ratios (HRs) and 95% CIs were calculated using the Cox regression model. The optimal cutoff values for the predictive models that were used to separate patients into low-risk and high-risk groups were generated using the survminer package. The prognostic or predictive accuracy of the signature and nomogram classifiers were assessed using a receiver operating characteristic curve analysis and by calculating the area under the curve (AUC).^21^ Additionally, a decision curve analysis (DCA) was performed to assess the clinical utility of the prediction model by quantifying the net benefits when different threshold probabilities were considered.^22,23^ For all analyses, *P* < .05 was considered statistically significant, and all tests were 2-tailed. The statistical analyses were performed using R version 3.6.1 (R Project for Statistical Computing). This study was registered with ClinicalTrials.gov (NCT04003558) and the Chinese Clinical Trail Registry (ChiCTR1900024020).

## Results

### Characteristics of the Cohorts

Table 1 and Table 2 show the clinical characteristics of the entire cohort (1214 participants; median [interquartile range {IQR}] age, 47 [42-55] years), development cohort (849 participants; median [IQR] age, 47 [42-56] years), and validation cohort (365 participants; median [IQR] age, 47 [41-54] years). The median (IQR) follow-up was 23.8 (15.3-37.7) months for patients in the entire cohort; the 1-year, 3-year, and 5-year DFS rates were 97.0% (95% CI, 96.0%-98.0%), 90.6% (95% CI, 88.5%-92.9%), and 85.4% (95% CI, 81.6%-89.4%), respectively; and the 1-year, 3-year, and 5-year OS rates were 99.9% (95% CI, 99.7%-100.0%), 97.9% (95% CI, 96.9%-99.0%), and 92.8% (95% CI, 89.4%-96.3%), respectively (eFigure 2 in the Supplement). Patients without ALNM were associated with significantly longer DFS (HR, 0.35; 95% CI, 0.22-0.56; *P* < .001) (eFigure 3A in the Supplement) and OS (HR, 0.16; 95% CI, 0.05-0.45; *P* < .001) (eFigure 3B in the Supplement) compared with patients with ALNM.

**Table 1.** Clinicopathologic Characteristics and Survival Rate of Patients in the Entire, Development, and Validation Cohorts

Characteristic	No. (%)		
	Entire cohort (N = 1214)	Development cohort (n = 849)	Validation cohort (n = 365)
Age, median (IQR), y	47 (42-55)	47 (42-56)	47 (41-54)
No. of tumors			
1	1043 (86.0)	725 (85.5)	318 (87.1)
>1	170 (14.0)	123 (14.5)	47 (12.9)
Tumor size, median (IQR), cm	2.3 (1.8-3.2)	2.4 (1.8-3.3)	2.3 (1.8-3.2)
Clinical T stage			
1	444 (36.6)	302 (35.6)	142 (38.9)
2	677 (55.8)	479 (56.5)	198 (54.2)
3	69 (5.7)	51 (6.0)	18 (4.9)
4	23 (1.9)	16 (1.9)	7 (1.9)
Clinical N stage			
0	775 (63.8)	551 (64.9)	224 (61.4)
1	388 (32.0)	259 (30.5)	129 (35.3)
2	44 (3.6)	35 (4.1)	9 (2.5)
3	7 (0.6)	4 (0.5)	3 (0.8)
Clinical TNM stage			
I	343 (28.3)	232 (27.4)	111 (30.4)
II	765 (63.1)	538 (63.4)	227 (62.2)
III	105 (8.7)	78 (9.2)	27 (7.4)
Histological type			
Invasive ductal carcinoma	1090 (89.8)	758 (89.3)	332 (91.0)
Invasive lobular carcinoma	51 (4.2)	35 (4.1)	16 (4.4)
Others	73 (6.0)	56 (6.6)	17 (4.7)
Histological grade			
1, low	33 (3.2)	18 (2.5)	15 (4.7)
2, intermediate	533 (51.6)	365 (51.3)	168 (52.2)
3, high	467 (45.2)	328 (46.1)	139 (43.2)
Pathological T stage			
1	596 (49.4)	408 (48.4)	188 (51.6)
2	545 (45.2)	387 (45.9)	158 (43.4)
3	52 (4.3)	38 (4.5)	14 (3.8)
4	14 (1.2)	10 (1.2)	4 (1.1)
Pathological N stage			
0	670 (55.2)	467 (55.0)	203 (55.6)
1	329 (27.1)	229 (27.0)	100 (27.4)
2	134 (11.0)	99 (11.7)	35 (9.6)
3	81 (6.7)	54 (6.4)	27 (7.4)
Pathological TNM stage			
I	372 (31.0)	259 (30.9)	113 (31.2)
II	592 (49.3)	407 (48.6)	185 (51.1)
III	236 (19.7)	172 (20.5)	64 (17.7)
ER status			
Negative	193 (16.0)	133 (15.7)	60 (16.6)
Positive	1016 (84.0)	714 (84.3)	302 (83.4)
PR status			
Negative	349 (28.9)	238 (28.1)	111 (30.7)
Positive	859 (71.1)	608 (71.9)	251 (69.3)
*ERBB2* status			
Negative	730 (68.4)	524 (70.1)	206 (64.4)
Positive	338 (31.6)	224 (29.9)	114 (35.6)
Ki-67 status			
<14	302 (25.1)	212 (25.1)	90 (24.9)
≥14	902 (74.9)	631 (74.9)	271 (75.1)
Molecular subtypes			
Luminal A	214 (18.4)	154 (18.9)	60 (17.1)
Luminal B	774 (66.5)	539 (66.2)	235 (67.1)
*ERBB2*-positive	92 (7.9)	64 (7.9)	28 (8.0)
Triple negative	84 (7.2)	57 (7.0)	27 (7.7)
Breast-conserving surgery			
Yes	506 (41.7)	343 (40.4)	163 (44.7)
No	707 (58.3)	505 (59.6)	202 (55.3)
Follow-up time, median (IQR), mo	23.8 (15.3-37.7)	23.7 (14.9-37.1)	23.9 (16.4-39.3)
DFS rate, % (95% CI)			
1-y	97.0 (96.0-98.0)	97.1 (95.9-98.3)	96.7 (94.8-98.6)
3-y	90.6 (88.5-92.9)	90.8 (88.2-93.4)	90.3 (86.2-94.7)
5-y	85.4 (81.6-89.4)	86.1 (81.8-90.7)	83.9 (76.8-91.7)
OS rate, % (95% CI)			
1-y	99.9 (99.7-100.0)	99.9 (99.6-100.0)	100.0 (100.0-100.0)
3-y	97.9 (96.9-99.0)	97.9 (96.6-99.2)	98.0 (96.1-100.0)
5-y	92.8 (89.4-96.3)	92.9 (88.7-97.4)	92.4 (86.8-98.5)

Abbreviations: DFS, disease-free survival; ER, estrogen receptor; IQR, interquartile range; OS, overall survival; PR, progesterone receptors; TNM, tumor-node-metastasis.

**Table 2.** Clinical Characteristics and Survival Rate of Patients With or Without Axillary Lymph Node Metastasis Across the Entire, Development, and Validation Cohorts

Characteristic	No. (%), by cohort and axillary lymph node status					
	Entire cohort (N = 1214)		Development cohort (n = 849)		Validation cohort (n = 365)	
	Negative (n = 670)	Positive (n = 544)	Negative (n = 467)	Positive (n = 382)	Negative (n = 203)	Positive (n = 162)
Age, median (IQR), y	48 (43-56)	46 (40-55)	48 (43-56)	46 (40-55)	48 (42-55)	46 (39-53)
No. of tumors						
1	584 (87.2)	459 (84.5)	400 (85.7)	325 (85.3)	184 (90.6)	134 (82.7)
>1	86 (12.8)	84 (15.5)	67 (14.3)	56 (14.7)	19 (9.4)	28 (17.3)
Tumor size, median (IQR), cm	2.1 (1.6-3.0)	2.7 (2.0-3.5)	2.2 (1.6-3.0)	2.6 (2.0-3.5)	2.1 (1.6-2.9)	2.7 (2.0-3.4)
Clinical T stage						
1	303 (45.3)	141 (25.9)	205 (44.0)	97 (25.4)	98 (48.3)	44 (27.2)
2	329 (49.2)	348 (64.0)	235 (50.4)	244 (63.9)	94 (46.3)	104 (64.2)
3	28 (4.2)	41 (7.5)	19 (4.1)	32 (8.4)	9 (4.4)	9 (5.6)
4	9 (1.3)	14 (2.6)	7 (1.5)	9 (2.4)	2 (1.0)	5 (3.1)
Clinical N stage						
0	549 (81.9)	226 (41.5)	391 (83.7)	160 (41.9)	158 (77.8)	66 (40.7)
1	118 (17.6)	270 (49.6)	74 (15.8)	185 (48.4)	44 (21.7)	85 (52.5)
2	2 (0.3)	42 (7.7)	2 (0.4)	33 (8.6)	0 (0.0)	9 (5.6)
3	1 (0.1)	6 (1.1)	0 (0.0)	4 (1.0)	1 (0.5)	2 (1.2)
Clinical TNM stage						
I	265 (39.6)	78 (14.3)	180 (38.6)	52 (13.6)	85 (41.9)	26 (16.0)
II	382 (57.1)	383 (70.4)	270 (57.9)	268 (70.2)	112 (55.2)	115 (71.0)
III	22 (3.3)	83 (15.3)	16 (3.4)	62 (16.2)	6 (3.0)	21 (13.0)
Histological type						
Invasive ductal carcinoma	597 (89.1)	493 (90.6)	410 (87.8)	348 (91.1)	187 (92.1)	145 (89.5)
Invasive lobular carcinoma	22 (3.3)	29 (5.3)	16 (3.4)	19 (5.0)	6 (3.0)	10 (6.2)
Others	51 (7.6)	22 (4.0)	41 (8.8)	15 (3.9)	10 (4.9)	7 (4.3)
Histological grade						
1, low	26 (4.6)	7 (1.5)	14 (3.7)	4 (1.2)	12 (6.5)	3 (2.2)
2, intermediate	303 (53.7)	230 (49.0)	211 (55.5)	154 (46.5)	92 (50.0)	76 (55.1)
3, high	235 (41.7)	232 (49.5)	155 (40.8)	173 (52.3)	80 (43.5)	59 (42.8)
Pathological T stage						
1	376 (56.4)	220 (40.7)	262 (56.5)	146 (38.5)	114 (56.2)	74 (46.0)
2	266 (39.9)	279 (51.7)	187 (40.3)	200 (52.8)	79 (38.9)	79 (49.1)
3	20 (3.0)	32 (5.9)	11 (2.4)	27 (7.1)	9 (4.4)	5 (3.1)
4	5 (0.7)	9 (1.7)	4 (0.9)	6 (1.6)	1 (0.5)	3 (1.9)
Pathological TNM stage						
I	372 (56.0)	0 (0.0)	259 (56.1)	0 (0.0)	113 (55.9)	0 (0.0)
II	287 (43.2)	305 (56.9)	199 (43.1)	208 (55.3)	88 (43.6)	97 (60.6)
III	5 (0.8)	231 (43.1)	4 (0.9)	168 (44.7)	1 (0.5)	63 (39.4)
ER status						
Negative	103 (15.5)	90 (16.6)	68 (14.6)	65 (17.0)	35 (17.4)	25 (15.5)
Positive	563 (84.5)	453 (83.4)	397 (85.4)	317 (83.0)	166 (82.6)	136 (84.5)
PR status						
Negative	193 (29.0)	156 (28.7)	130 (28.0)	108 (28.3)	63 (31.3)	48 (29.8)
Positive	472 (71.0)	387 (71.3)	334 (72.0)	274 (71.7)	138 (68.7)	113 (70.2)
*ERBB2* status						
Negative	424 (71.7)	306 (64.2)	296 (71.5)	228 (68.3)	128 (72.3)	78 (54.5)
Positive	167 (28.3)	171 (35.8)	118 (28.5)	106 (31.7)	49 (27.7)	65 (45.5)
Ki-67 status						
<14	179 (27.0)	123 (22.7)	125 (27.0)	87 (22.9)	54 (27.0)	36 (22.4)
≥14	484 (73.0)	418 (77.3)	338 (73.0)	293 (77.1)	146 (73.0)	125 (77.6)
Molecular subtypes						
Luminal A	134 (20.9)	80 (15.3)	95 (21.2)	59 (16.1)	39 (20.1)	21 (13.5)
Luminal B	412 (64.2)	362 (69.3)	291 (65.0)	248 (67.8)	121 (62.4)	114 (73.1)
*ERBB2*-positive	45 (7.0)	47 (9.0)	33 (7.4)	31 (8.5)	12 (6.2)	16 (10.3)
Triple negative	51 (7.9)	33 (6.3)	29 (6.5)	28 (7.7)	22 (11.3)	5 (3.2)
Breast-conserving surgery						
Yes	351 (52.5)	356 (65.4)	248 (53.2)	257 (67.3)	103 (50.7)	99 (61.1)
No	318 (47.5)	188 (34.6)	218 (46.8)	125 (32.7)	100 (49.3)	63 (38.9)
Follow-up time, median (IQR), mo	23.8 (15.1-36.9)	23.8 (15.5-38.8)	23.3 (14.6-36.2)	24.0 (15.4-38.3)	24.1 (16.5-40.1)	22.5 (16.1-38.9)
DFS rate, % (95% CI)						
1-y	98.8 (98.0-99.7)	94.7 (92.7-96.7)	98.8 (97.7-99.8)	95.1 (92.8-97.4)	98.9 (97.5-100.0)	93.9 (90.1-97.8)
3-y	94.5 (92.2-97.0)	86.0 (82.2-90.0)	94.4 (91.6-97.3)	86.5 (82.1-91.1)	94.9 (90.6-99.4)	84.5 (77.2-92.6)
5-y	91.0 (86.4-95.8)	79.1 (73.1-85.5)	91.2 (86.3-96.5)	80.6 (73.7-88.2)	90.4 (81.3-100.0)	74.6 (62.9-88.5)
OS rate, % (95% CI)						
1-y	100.0 (100.0-100.0)	99.8 (99.4-100.0)	100.0 (100.0-100.0)	99.7 (99.2-100.0)	100.0 (100.0-100.0)	100.0 (100.0-100.0)
3-y	99.5 (98.7-100.0)	96.1 (94.0-98.3)	99.6 (98.9-100.0)	95.9 (93.3-98.5)	99.2 (97.6-100.0)	96.5 (92.6-100.0)
5-y	97.7 (95.1-100.0)	87.7 (81.6-94.2)	96.7 (92.7-100.0)	89.3 (82.4-96.7)	99.2 (97.6-100.0)	83.6 (71.8-97.4)

Abbreviations: DFS, disease-free survival; ER, estrogen receptor; IQR, interquartile range; OS, overall survival; PR, progesterone receptors; TNM, tumor–node–metastasis.

### Radiomic Signature for ALNM Prediction

An unsupervised consensus clustering analysis using 2589 radiomic features from the ALN T1+C, T2WI, and DWI-ADC sequences in the entire cohort was performed first. Two main radiomic clusters associated with significant differences in ALNM were found (OR, 1.79; 95% CI, 1.30-2.46; *P* < .001) (eFigure 4 in the Supplement). In the development and validation cohorts, the LASSO–logistic regression model resulted in AUCs of 0.88 and 0.85, respectively; the LASSO-SVM model resulted in AUCs of 0.86 and 0.74, respectively; the random forest–logistic regression model resulted in AUCs of 0.94 and 0.53, respectively; and the random forest–SVM model resulted in AUCs of 0.86 and 0.75, respectively, for ALNM radiomic signature. The LASSO–logistic regression model was selected as the basic model with best performance for ALN radiomic signature between the development and validation cohorts (eFigure 5, eFigure 6, and eTable 3 in the Supplement). The results of tumor radiomic signature (AUCs, 0.86 and 0.60 in the development and validation cohorts, respectively) or combination of tumor and ALN radiomic signature (AUCs, 0.90 and 0.71 in the development and validation cohorts, respectively) were no better than ALN radiomic signature alone for ALNM prediction. More detailed results are summarized in eTable 4 and eTable 5 in the Supplement.

### Clinical-Radiomic Nomogram for ALNM Prediction

**Figure 2.**
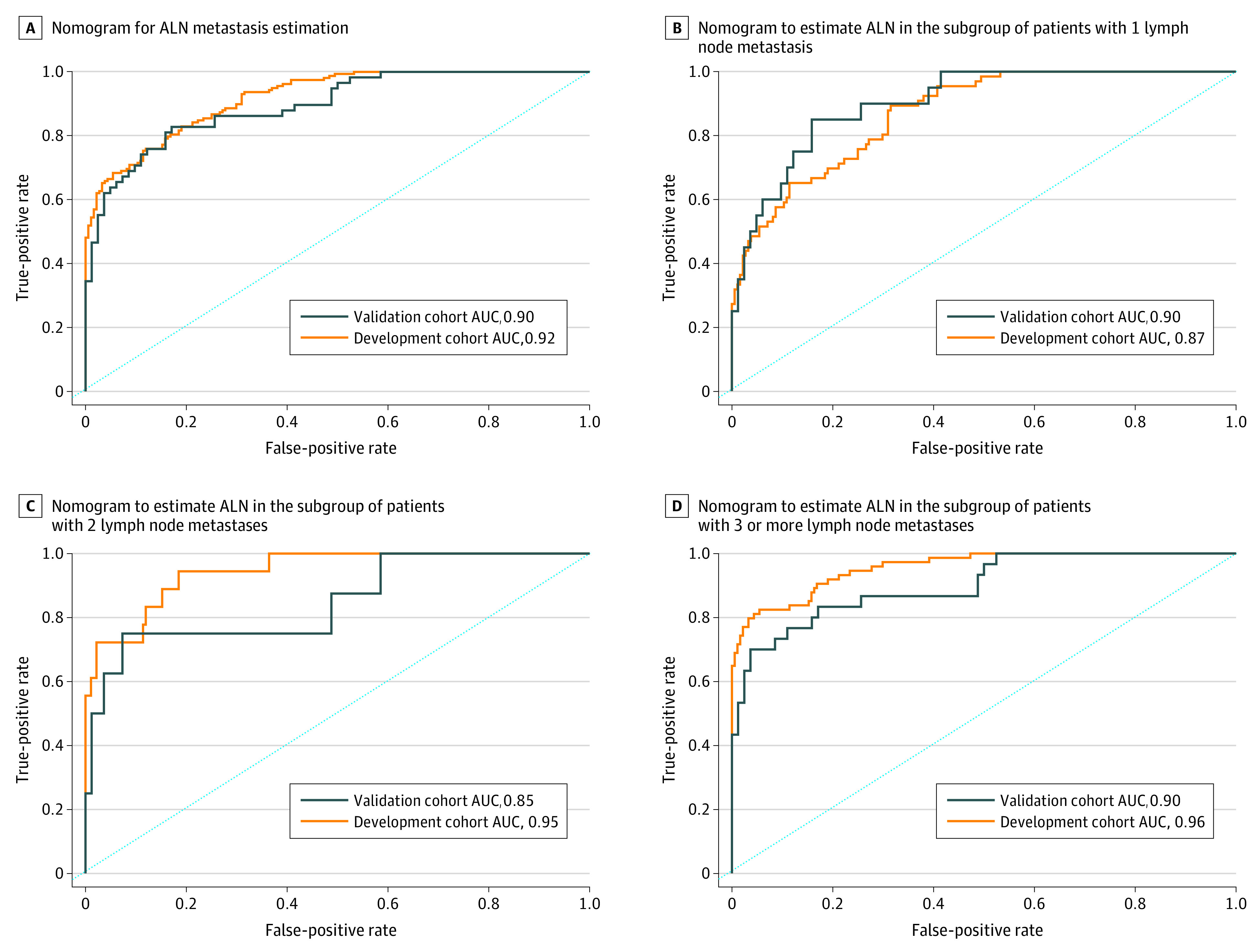
Clinical-Radiomic Nomogram for Predicting Lymph Node Metastasis ALN indicates axillary lymph node; AUC, area under the receiver operating characteristic curve.

eTable 6 in the Supplement summarizes the univariable analyses of ALNM status in relation to the characteristics of the cohorts. Young age, high histological grade, high clinical T and N stage, and *ERBB2* (formerly known as *HER2*)–positive status showed significant positive associations with ALNM. The clinical signature that incorporated the significant factors showed AUCs of 0.77 and 0.71 in the development and validation cohorts, respectively (eFigure 7 in the Supplement). The multivariable analysis identified clinical and radiomic signatures as independent predictors of ALNM in patients (eTable 7 in the Supplement). The clinical-radiomic nomogram (eFigure 8 and eTable 3 in the Supplement) that incorporated these clinical and radiomic signatures resulted in stronger associations with ALNM in the development (AUC, 0.92) and validation (AUC, 0.90) cohorts (Figure 2A).

The effectiveness of the clinical-radiomic nomogram was validated in the SYSMH/SYSUCC cohort (AUC, 0.92) and the SYSUTH/SMUSH cohort (AUC, 0.90) (eFigure 9 in the Supplement). ALNM status also showed a good predictive capacity for the development and validation cohorts in the subgroups of patients with 1 lymph node metastasis (AUCs of 0.87 and 0.90, respectively), those with 2 (AUCs of 0.95 and 0.85, respectively), and those with 3 or more (AUCs of 0.96 and 0.90, respectively) (Figure 2B, Figure 2C, and Figure 2D), and in patients in the luminal A (AUCs of 0.91 and 0.85, respectively), luminal B (AUCs of 0.92 and 0.90, respectively), *ERBB2*-positive (AUCs of 0.97 and 0.83, respectively), and triple-negative breast cancer (TNBC) (AUCs of 0.92 and NA, respectively) groups (eFigure 10 in the Supplement). The DCA indicated that if the threshold probability in clinical decision was greater than 10% for predicting ALN metastases, the use of the clinical-radiomic nomogram added more benefit than either the clinical or radiomic signature alone (eFigure 11 in the Supplement).

### Radiomic Signature for DFS Prediction

A total of 2589 features from the tumor T1+C, T2WI, and DWI-ADC sequences underwent unsupervised consensus clustering for the entire cohort to identify 2 main radiomic clusters associated with the significant difference in DFS (HR, 0.45; 95% CI, 0.26-0.78; *P* = .004) (eFigure 12 in the Supplement). The LASSO–Cox regression model resulted in AUCs of 0.83, 0.82, and 0.78 for 1-year, 2-year, and 3-year DFS prediction, respectively, in the development cohort, and 0.54, 0.64, and 0.66, respectively, in the validation cohort. The random forest–Cox regression model resulted in AUCs of 0.80, 0.83, and 0.81 for 1-year, 2-year, 3-year DFS prediction, respectively, in the development cohort and 0.68, 0.74, and 0.73, respectively, in the validation cohort. The random forest–Cox regression model was selected as the basic model with more consistent performance for DFS prediction between the development and validation cohorts, and the radiomic signature discriminated high-risk from low-risk patients in the development cohort (HR, 0.09; 95% CI, 0.05-0.17; *P* < .001) and the validation cohort (HR, 0.31; 95% CI, 0.10-1.00; *P* = .045) (eFigures 13-16, eTable 8 and eTable 9 in the Supplement).

### Clinical-Radiomic Nomogram for DFS Prediction

eFigure 17 in the Supplement summarizes the univariable analyses results in the development and validation cohorts. An increased number of tumors, high histological grade, high pathological TNM stage, progesterone receptor–negative status, high Ki-67 status, and nonbreast conversing surgery were significantly associated with poor DFS, and these factors were used to calculate the clinical signature. The clinical signature discriminated high-risk from low risk patients in the development cohort (HR, 0.10; 95% CI, 0.05-0.19; *P* < .001) and the validation cohort (HR, 0.18; 95% CI, 0.07-0.45; *P* < .001). In addition, it showed AUCs for 1-year, 2-year, and 3-year DFS of 0.83, 0.83, and 0.85, respectively, in the development cohort and 0.76, 0.78, and 0.80, respectively, in the validation cohort (eFigure 18 and eTable 9 in the Supplement).

**Figure 3.**
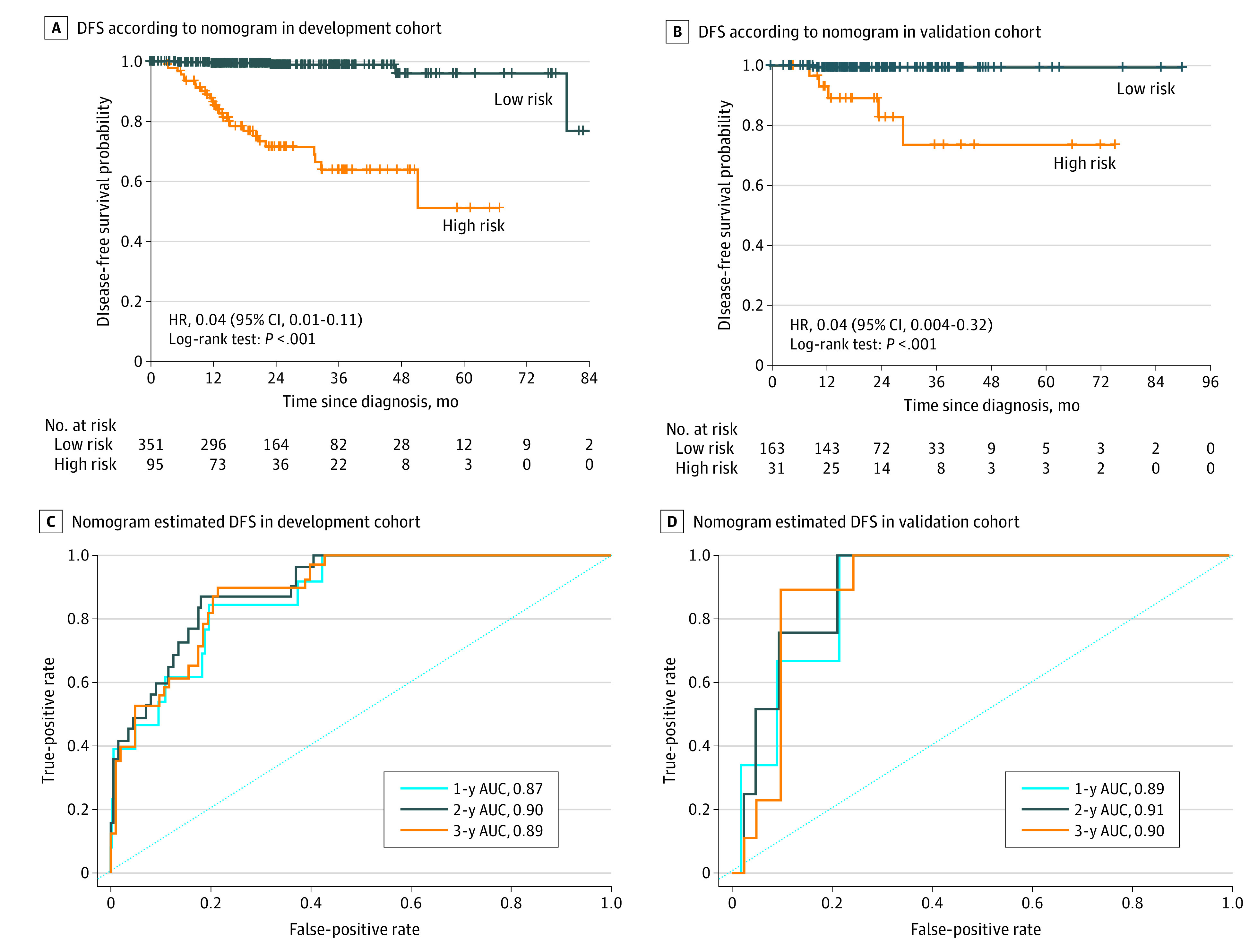
Clinical-Radiomic Nomogram for Predicting Disease-Free Survival (DFS) AUC indicates area under the receiver operating characteristic curve; HR, hazard ratio.

The multivariable analysis identified significant clinical and radiomic signatures as independently associated with DFS (eTable 10 in the Supplement). The clinical-radiomic nomogram discriminated high-risk from low risk patients in the development cohort (HR, 0.04; 95% CI, 0.01-0.11; *P* < .001) and the validation cohort (HR, 0.04; 95% CI, 0.004-0.32; *P* < .001). The clinical-radiomic nomogram showed a stronger correlation with DFS in the development cohort (AUCs for 1-year, 2-year, and 3-year DFS of 0.87, 0.90, and 0.89, respectively) and the validation cohort (AUCs for 1-year, 2-year, and 3-year DFS of 0.89, 0.91, and 0.90, respectively) compared with the clinical or radiomic signature alone (Figure 3; eFigure 19 and eTable 9 in the Supplement).

The clinical-radiomic nomogram also discriminated high-risk from low risk patients in the SYSMH and SYSUCC cohorts (HR, 0.03; 95% CI, 0.01-0.10; *P* < .001) and the SYSUTH and SMUSH cohorts (HR, 0.06; 95% CI, 0.01-0.26; *P* < .001). It also predicted the 1-year, 2-year, and 3-year DFS in the SYSMH and SYSUCC cohort (AUCs of 0.87, 0.89, and 0.89, respectively) and the SYSUTH and SMUSH cohorts (AUCs of 0.90, 0.94, and 0.92, respectively) (eFigure 20 in the Supplement). In addition, it discriminated high-risk from low-risk patients in the subgroups of luminal A (AUCs for 1-year, 2-year, and 3-year DFS of 0.98, 0.97, and 0.94, respectively), luminal B (AUCs for 1-year, 2-year, and 3-year DFS of 0.86, 0.88, and 0.88, respectively), *ERBB*2-positive (AUCs for 1-year, 2-year, and 3-year DFS of 0.87, 0.92, and 0.92, respectively), and TNBC (AUCs for 1-year, 2-year, and 3-year DFS of 0.75, 0.90, and 0.90, respectively) patients (eFigure 21 in the Supplement). The DCA indicated that if the threshold probability of the clinical decision was greater than 5% for predicting DFS, using the clinical-radiomic nomogram added more benefit than either the clinical or radiomic signature alone (eFigure 22 in the Supplement).

## Discussion

In this study, novel nomograms were developed and validated; they incorporated radiomic and clinical signatures to provide an easy-to-use and individualized prediction of ALNM and risk of disease recurrence in patients with early-stage breast cancer. The clinical-radiomic nomogram successfully stratified early-stage breast cancer patients according to their risk of ALNM. This technique displayed a greater net benefit and was able to reduce the number of unnecessary ALNDs compared with use of radiologists’ diagnosis to select patients for sentinel lymph node biopsy at all threshold probabilities. The patients stratified by the combined radiomic and clinical signature were classified into low-risk and high-risk groups for DFS in advance, which greatly improved the ability to predict the risk of DFS. This strategy may have clinical implications for individualized follow-up and for guiding therapeutic strategies.

The determination of negative ALNM status was clinically meaningful for prognosis prediction. The results of this study showed a significant DFS and OS difference between patients without or with ALN metastasis, suggesting that an understanding of the radiomic features of the ALNM subtypes could aid in improvements in patient prognoses. This determination is also helpful for preventing the implementation of ALND, which can likely produce complications, such as edema, pain, and paresthesia.^24,25^ Two previous studies^26,27^ tried to use the MRI radiomic signature to predict ALNM status to develop a more precise and noninvasive preoperative evaluation of the ALN status, but several limitations restricted their clinical usefulness in clinical decision-making. They included small sample sizes of only 62 and 163 patients from a single center, which likely lowered the generalizability and clinical applicability. In addition, the MRI radiomics were based on only single a T1-weighted sequence, which might be inadequate to reflect tumor heterogeneity. Compared with these studies, the data sets in this study were, to our knowledge, the largest to date, involving more than 1000 patients from 4 medical institutions. This study also used additional T2WI and DWI-ADC sequences that more robustly interpreted the significance of the radiomics. Additionally, previous radiomic studies focused on using features from the intertumoral and peritumoral regions^26,27^ without considering the distinct differences in the tumor microenvironment between the ALN and primary tumor site. This study used the largest data set to date that we know of to demonstrate the feasibility of identifying predictive radiomic features from axillary regions, including both sentinel lymph node and non–sentinel lymph node sites, considering the possibility of non–sentinel lymph node metastasis that would not be detected in normal routine.

Moreover, nomograms that incorporate the radiomic signature and clinical signature covariates were developed and validated in this study, providing a more precise multiomics method for predicting the preoperative status of ALNM. In the subgroup analysis, the diagnostic performance was further shown to distinguish patients with 1 lymph node metastasis, those with 2, and those with 3 or more. A 2020 study showed good performance of a nomogram for predicting preoperative ALN involvement based on ultrasonographic radiomic signatures.^11^ It is suggested that future studies should focus on comparing predictive values from different imaging approaches, such as MRI and ultrasonography, using artificial intelligence technology.

The development of artificial intelligence–based radiomics in the medical field has allowed for the identification of low-risk and high-risk patients with early-stage breast cancer and has provided useful clinical decision-making guidance. A previous study that recruited 294 patients with invasive breast cancer found an encouraging association between MRI radiomics and DFS.^28^ Consistent with this finding, this multicenter study had an increased recruitment by 321.1% and confirmed the predictive abilities of MRI radiomics for DFS. In current clinical practice, multigene-based expression assays, such as the 70-gene expression profile and the 21-gene recurrence score, are currently being used to predict the likelihood of recurrence in patient subsets who have had hormone receptor–positive, *ERBB2*-negative early-stage breast tumors.^5,6^ It is suggested that the combination of radiomics and genomics might better guide clinical decision-making.

### Limitations

This study has several limitations. First, the heterogeneity of MR versions exists across and within centers due to the retrospective nature of the study; therefore, each patient scan was normalized to get a standard distribution of image intensities to reduce heterogeneity, and all patients were randomly grouped to reduce the heterogeneity between the development and validation cohorts. Second, some patients’ MRI (T1+C, T2WI, or DWI-ADC) sequences were somewhat scattered based on the retrospective nature of the study. Third, the median (IQR) follow-up was 23.8 (15.3-37.6) months for patients in the entire cohort. Future prospective trials with a longer follow-up are needed to validate our nomogram and to develop and validate radiomics-based signatures for individual OS assessment in patients with early-stage breast cancer. Additionally, due to a lack of available data, we were unable to further consider the genetic signatures comprising genomics, transcriptomics, and tumor mutation burden to enhance the accuracy and interpretability of this model, and the uncertainty regarding the potential mechanisms driving the interaction between radiomic features and tumor microenvironment warrants further investigation. Additionally, multiple models were evaluated in the validation set, a form of learning on held-out data, where model performance was inconsistent or overfitting may have occurred; we selected the single best-performing model for further analysis, but more independent prospective external validations and more stable algorithms should be validated.

## Conclusions

This study found that the radiomic and clinical signature tool could effectively classify patients into groups with different risks of ALNM and DFS, and the clinical-radiomic nomograms were more strongly associated with the ALNM and DFS prediction than other models. This knowledge can assist clinicians in directing personalized therapeutic regimen selections for patients with early-stage breast cancer. However, the results of this study will require further calibration and validation using a high-quality prospective study.

## Notes

Supplement. **eAppendix. **Inclusion and Exclusion Criteria and Outcomes **eFigure 1. **Patient Recruitment and Study Design **eFigure 2. **Kaplan-Meier Survival Analyses of DFS and OS in the Entire Cohort **eFigure 3. **Kaplan-Meier Survival Analysis Stratified by ALN Metastasis Status in the Entire Cohort **eFigure 4. **Unsupervised Clustering Analysis of MRI Multisequence Data of Axillary Region of Interest **eFigure 5. **Identification of Essential Radiomic Features of T1+C, T2WI, and DWI-ADC Sequences with LASSO Regression Analysis **eFigure 6. **ROC Curves of Radiomic Signature for ALN Metastasis Prediction in the Development and Validation Cohorts **eFigure 7. **ROC Curves of Clinical Signature for ALN Metastasis Prediction in the Development and Validation Cohorts **eFigure 8. **Clinical-Radiomic Nomogram Developed in the Development Cohort to Predict Axillary Lymph Node Metastasis **eFigure 9. **ROC Curves of Clinical-Radiomic Nomogram for ALN Metastasis Prediction in Different Institutions **eFigure 10. **ROC Curves of Clinical-Radiomic Nomogram for ALN Metastasis Prediction in Different Molecular Subtype **eFigure 11. **Decision Curve Analysis for ALN Metastasis Prediction in the Development and Validation Cohorts **eFigure 12. **Unsupervised Clustering Analysis of MRI Multisequence Data of Primary Breast Tumors **eFigure 13. **ROC Curves of T1+C Sequence Radiomic Signature for DFS Prediction **eFigure 14. **ROC Curves of T2WI Sequence Radiomic Signature for DFS Prediction **eFigure 15. **ROC Curves of DWI-ADC Sequence Radiomic Signature for DFS Prediction **eFigure 16. **The Performance of Radiomic Signature Combining T1+C, T2WI, and DWI-ADC Features in Predicting DFS **eFigure 17. **Univariate Association of Clinicopathological Characteristics with DFS **eFigure 18. **The Performance of Clinical Signature in Predicting DFS **eFigure 19. **Clinical-Radiomic Nomogram Developed in the Development Cohort to Predict 1-Year, 2-Year, and 3-Year DFS **eFigure 20. **ROC curves of Clinical-Radiomic Nomogram for 1-Year, 2-Year, and 3-Year DFS Prediction in Different Institutions **eFigure 21. **ROC Curves of Clinical-Radiomic Nomogram for 1-Year, 2-Year, and 3-Year DFS Prediction in Different Molecular Subtypes in the Entire Cohort **eFigure 22. **Decision Curve Analysis for DFS Prediction in the Development and Validation Cohorts **eTable 1. **Information of 4 Institutions in This Study **eTable 2. **MR Scanning Parameters for the Patients **eTable 3. **The Performance to Predict Axillary Lymph Node Metastasis among Signatures Constructed by Different Algorithms **eTable 4. **The Performance of Different Regions of Interest Constructed for Axillary Lymph Node Metastasis Prediction **eTable 5. **Essential Radiomic Features and Formula Composition for ALN Metastasis Prediction **eTable 6. **Univariable Analysis of Axillary Lymph Node Status in Relation to Clinicopathologic Characteristics **eTable 7. **Multivariate Analysis of Axillary Lymph Node Metastasis in Relation to Clinical Signature and Radiomic Signature in the Development Cohort **eTable 8. **The Performance of Each Signature Constructed With Different Algorithm for DFS Prediction **eTable 9. **Essential Radiomic Features and Formula Composition for DFS Prediction **eTable 10. **Multivariate Analysis of Clinical Signature and Radiomic Signature with DFS in the Development Cohort
