# Supplementary material for: Development and Validation of a Preoperative Magnetic Resonance Imaging Radiomics–Based Signature to Predict Axillary Lymph Node Metastasis and Disease-Free Survival in Patients With Early-Stage Breast Cancer
Source: JAMA Netw Open. 2020 Dec 8;3(12):e2028086. doi: 10.1001/jamanetworkopen.2020.28086 (PMC7724560; doi:10.1001/jamanetworkopen.2020.28086)
Supplement: Supplement. — eAppendix. Inclusion and Exclusion Criteria and Outcomes eFigure 1. Patient Recruitment and Study Design eFigure 2. Kaplan-Meier Survival Analyses of DFS and OS in the Entire Cohort eFigure 3. Kaplan-Meier Survival Analysis Stratified by ALN Metastasis Status in the Entire Cohort eFigure 4. Unsupervised Clustering Analysis of MRI Multisequence Data of Axillary Region of Interest eFigure 5. Identification of Essential Radiomic Features of T1+C, T2WI, and DWI-ADC Sequences with LASSO Regression Analysis eFigure 6. ROC Curves of Radiomic Signature for ALN Metastasis Prediction in the Development and Validation Cohorts eFigure 7. ROC Curves of Clinical Signature for ALN Metastasis Prediction in the Development and Validation Cohorts eFigure 8. Clinical-Radiomic Nomogram Developed in the Development Cohort to Predict Axillary Lymph Node Metastasis eFigure 9. ROC Curves of Clinical-Radiomic Nomogram for ALN Metastasis Prediction in Different Institutions eFigure 10. ROC Curves of Clinical-Radiomic Nomogram for ALN Metastasis Prediction in Different Molecular Subtype eFigure 11. Decision Curve Analysis for ALN Metastasis Prediction in the Development and Validation Cohorts eFigure 12. Unsupervised Clustering Analysis of MRI Multisequence Data of Primary Breast Tumors eFigure 13. ROC Curves of T1+C Sequence Radiomic Signature for DFS Prediction eFigure 14. ROC Curves of T2WI Sequence Radiomic Signature for DFS Prediction eFigure 15. ROC Curves of DWI-ADC Sequence Radiomic Signature for DFS Prediction eFigure 16. The Performance of Radiomic Signature Combining T1+C, T2WI, and DWI-ADC Features in Predicting DFS eFigure 17. Univariate Association of Clinicopathological Characteristics with DFS eFigure 18. The Performance of Clinical Signature in Predicting DFS eFigure 19. Clinical-Radiomic Nomogram Developed in the Development Cohort to Predict 1-Year, 2-Year, and 3-Year DFS eFigure 20. ROC curves of Clinical-Radiomic Nomogram for 1-Year, 2-Year, and 3-Year DFS Prediction in D [file jamanetwopen-e2028086-s001.pdf]

Supplemental Online Content

Yu Y, Tan Y, Xie C, et al. Development and validation of a preoperative magnetic resonance imaging radiomics–based signature to predict axillary lymph node metastasis and disease-free survival in patients with early-stage breast cancer. *JAMA Netw Open*. 2020;3(12):e2028086. doi:10.1001/jamanetworkopen.2020.28086

eAppendix. Inclusion and Exclusion Criteria and Outcomes

eFigure 1. Patient Recruitment and Study Design

eFigure 2. Kaplan-Meier Survival Analyses of DFS and OS in the Entire Cohort

eFigure 3. Kaplan-Meier Survival Analysis Stratified by ALN Metastasis Status in the Entire Cohort

eFigure 4. Unsupervised Clustering Analysis of MRI Multisequence Data of Axillary Region of Interest

eFigure 5. Identification of Essential Radiomic Features of T1+C, T2WI, and DWI-ADC Sequences with LASSO Regression Analysis

eFigure 6. ROC Curves of Radiomic Signature for ALN Metastasis Prediction in the Development and Validation Cohorts

eFigure 7. ROC Curves of Clinical Signature for ALN Metastasis Prediction in the Development and Validation Cohorts

eFigure 8. Clinical-Radiomic Nomogram Developed in the Development Cohort to Predict Axillary Lymph Node Metastasis

eFigure 9. ROC Curves of Clinical-Radiomic Nomogram for ALN Mmetastasis Prediction in Different Institutions

eFigure 10. ROC Curves of Clinical-Radiomic Nomogram for ALN Metastasis Prediction in Different Molecular Subtype

eFigure 11. Decision Curve Analysis for ALN Metastasis Prediction in the Development and Validation Cohorts

eFigure 12. Unsupervised Clustering Analysis of MRI Multisequence Data of Primary Breast Tumors

eFigure 13. ROC Curves of T1+C Sequence Radiomic Signature for DFS Prediction

eFigure 14. ROC Curves of T2WI Sequence Radiomic Signature for DFS Prediction

eFigure 15. ROC Curves of DWI-ADC Ssequence Radiomic Signature for DFS Prediction

© 2020 Yu Y et al. *JAMA Network Open.*

eFigure 16. The Performance of Radiomic Signature Combining T1+C, T2WI, and DWI-ADC Features in Predicting DFS

eFigure 17. Univariate Association of Clinicopathological Characteristics with DFS

eFigure 18. The Performance of Clinical Signature in Predicting DFS

eFigure 19. Clinical-Radiomic Nomogram Developed in the Development Cohort to Predict 1-Year, 2-Year, and 3-Year DFS

eFigure 20. ROC curves of Clinical-Radiomic Nomogram for 1-Year, 2-Year, and 3-Year DFS Prediction in Different Institutions

eFigure 21. ROC Curves of Clinical-Radiomic Nomogram for 1-Year, 2-Year, and 3-Year DFS Prediction in Different Molecular Subtypes in the Entire Cohort

eFigure 22. Decision Curve Analysis for DFS Prediction in the Development and Validation Cohorts

eTable 1. Information of 4 Institutions in This Study

eTable 2. MR Scanning Parameters for the Patients

eTable 3. The Performance to Predict Axillary Lymph Node Metastasis Among Signatures Constructed by Different Algorithms

eTable 4. The Performance of Different Region of Interest Constructed for Axillary Lymph Node Metastasis Prediction

eTable 5. Essential Radiomic Features and Formula Composition for ALN Metastasis Prediction

eTable 6. Univariable Analysis of Axillary Lymph Node Status in Relation to Clinicopathologic Characteristics

eTable 7. Multivariate Analysis of Axillary Lymph Node Metastasis in Relation to Clinical Signature and Radiomic Signature in the Development Cohort

eTable 8. The Performance of Each Signature Constructed With Different Algorithm for DFS Prediction

eTable 9. Essential Radiomic Features and Formula Composition for DFS Prediction

eTable 10. Multivariate Analysis of Clinical Signature and Radiomic Signature with DFS in the Development Cohort

This supplemental material has been provided by the authors to give readers additional information about their work.

© 2020 Yu Y et al. *JAMA Network Open.*

eAppendix. Inclusion and Exclusion Criteria and Outcomes

The inclusion criteria were as follows: (i) female patients staged I-III and aged at least 18 years; (ii) patients had histologically confirmed unilateral primary breast cancer without distant metastasis; (iii) patients who had been treated with surgery and sentinel lymph node biopsy or ALN dissection (ALND), and had been pathologically examined to determine axillary lymph node (ALN) status; (iv) preoperative dynamic contrast–enhanced magnetic resonance imaging (DCE-MRI) scan of breast tumor and/or ALN were conducted, including contrast-enhanced T1-weighted imaging (T1+C), T2-weighted imaging (T2WI), and diffusion-weighted imaging quantitatively measured apparent diffusion coefficients (DWI-ADC); the post-neoadjuvant therapy MRI was selected when the patients undergo preoperative systemic treatment. Key exclusion criteria were: (i) patients underwent biopsy at an external institution and pathological results were not available; (ii) patients previously had non-breast cancer without complete remission for more than 3 years; (iii) insufficient DCE-MRI quality to obtain measurements; and (iv) the correlation between the breast tumor and ALN in DCE-MRI and postoperative pathologic examination was uncertain. All patients were staged according to the 8th edition of the American Joint Committee on Cancer Staging Manual.[1]

The primary endpoints included ALNM and disease-free survival (DFS). DFS were defined as the time from the diagnosis of breast cancer and ALND surgery to the first relapse at any site, or confirmed time of metastatic disease, or death due to any other cause, or the date of the last follow-up visit, whichever occurred first. Secondary endpoint included overall survival (OS), which was defined as the time from diagnosis of breast cancer to death from any cause.

eReferences

1. Amin MB, Edge S, Greene F, et al. *AJCC cancer staging manual, 8th edn*. New York, NY: Springer; 2016.

© 2020 Yu Y et al. *JAMA Network Open.*

.**eFigure 1.** Patient Recruitment and Study Design

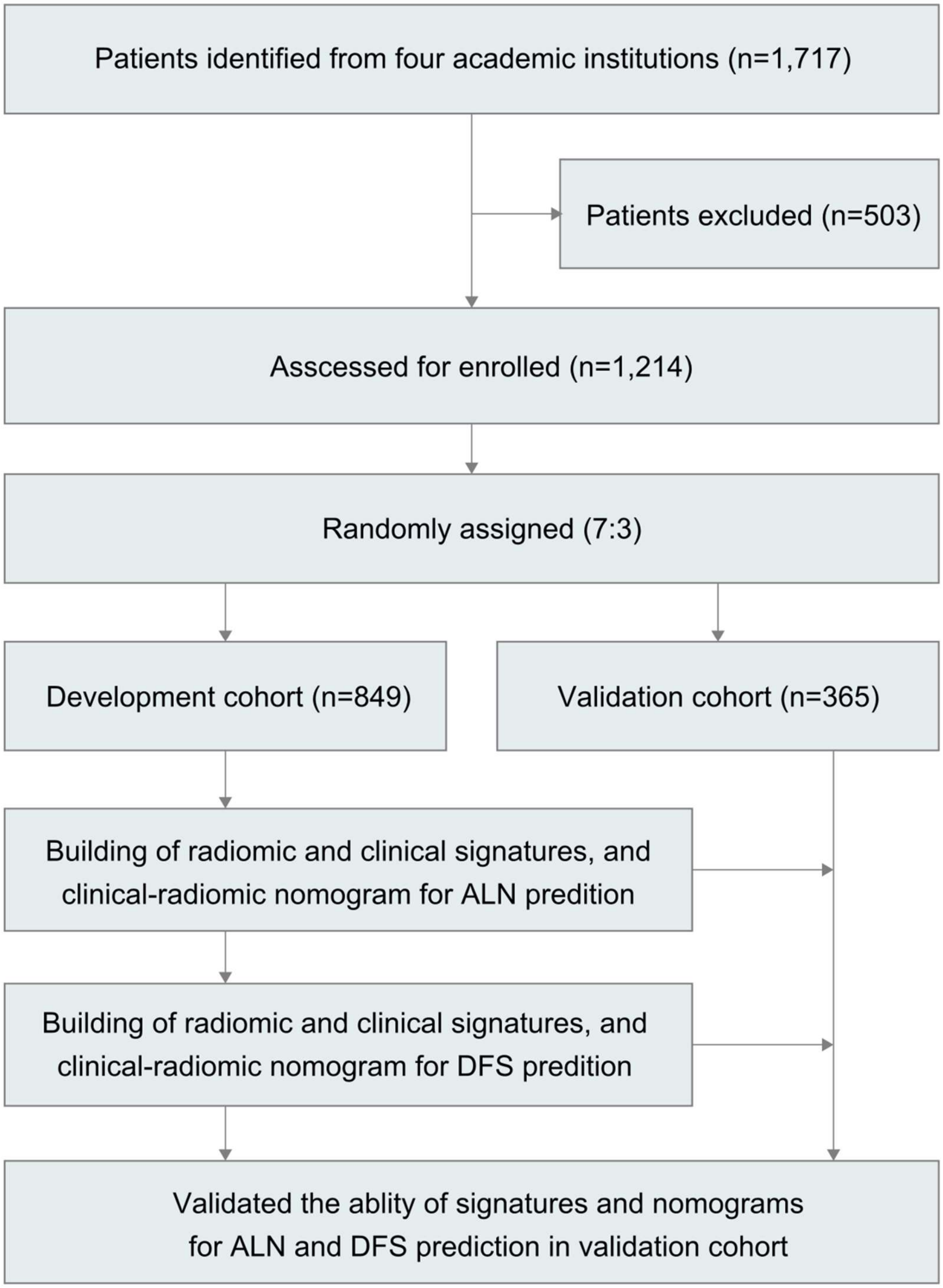

In total, 1214 patients with preoperative magnetic resonance imaging from four academic institutions were enrolled in this study for model construction and validation. DFS, disease-free survival; ALN, axillary lymph node.

© 2020 Yu Y et al. *JAMA Network Open*.

eFigure 2. Kaplan-Meier Survival Analyses of DFS and OS in the Entire Cohort

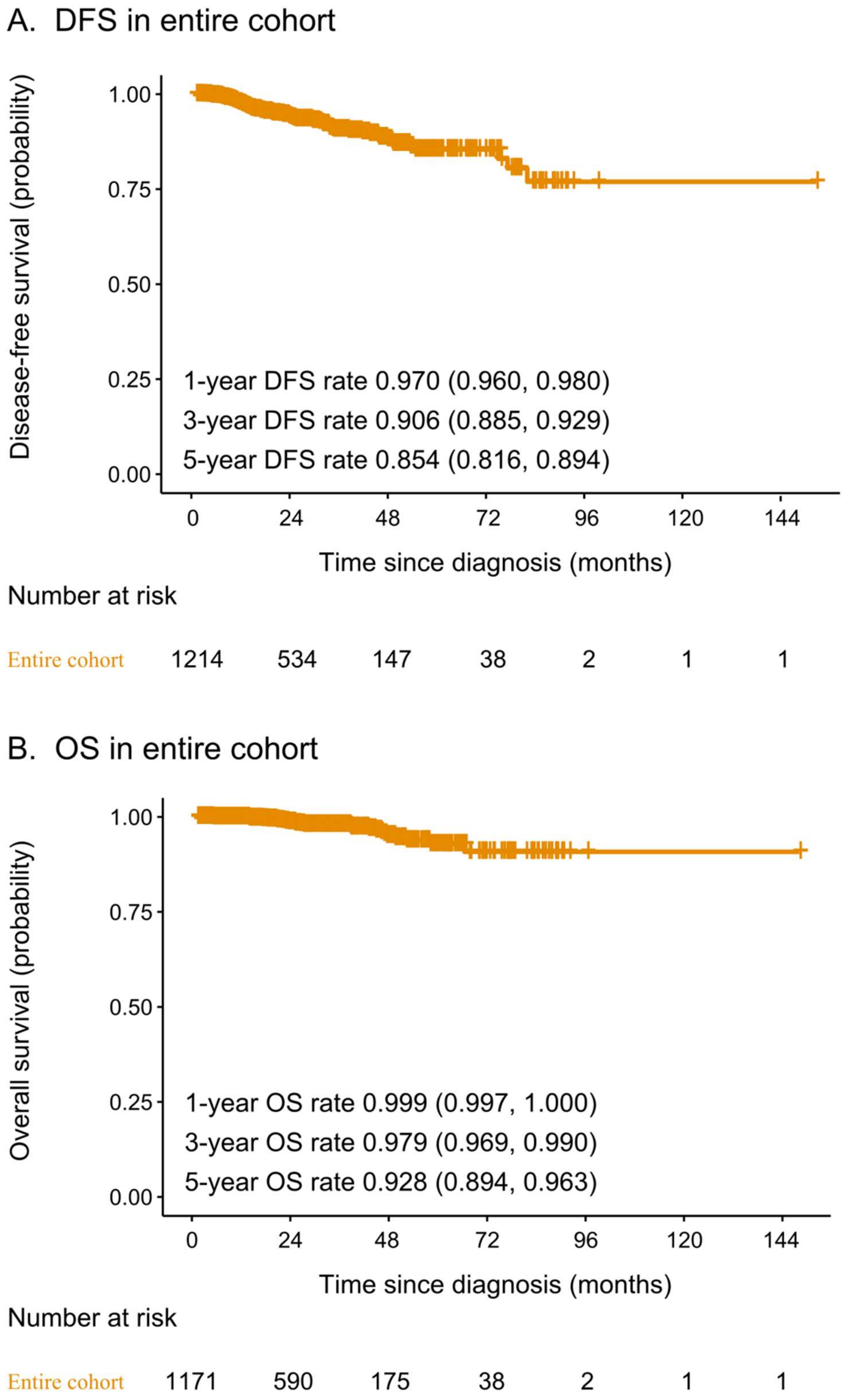

DFS (A) and OS (B) for patients in the entire cohort. DFS, disease-free survival; OS, overall survival; HR, hazard ratio; CI, confidence interval.

© 2020 Yu Y et al. *JAMA Network Open.*

eFigure 3. Kaplan-Meier Survival Analysis Stratified by ALN Metastasis Status in the Entire Cohort

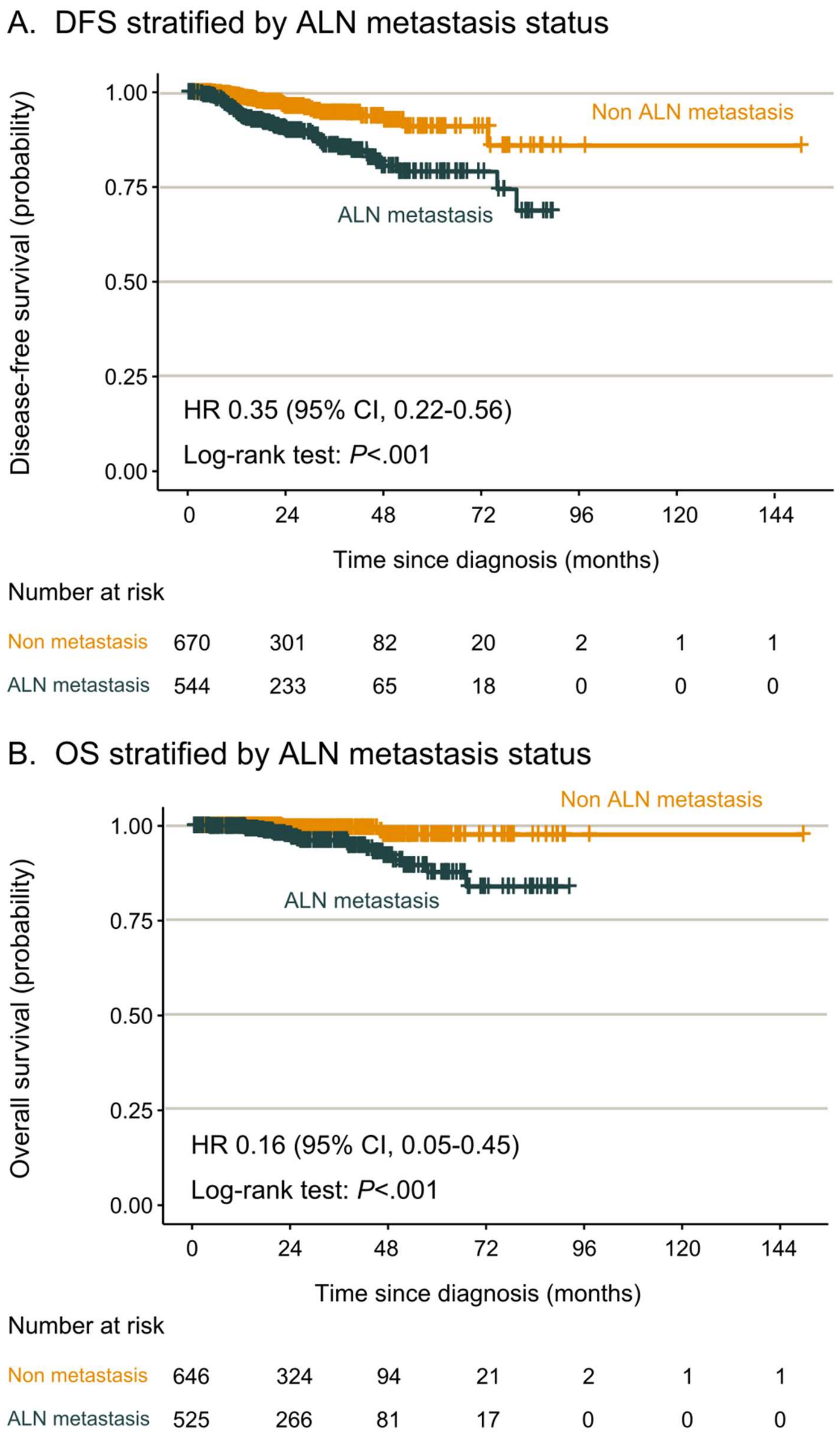

(A) DFS stratified by ALN metastasis status for patients in the entire cohort. (B) OS stratified by ALN metastasis status for patients in the entire cohort. DFS, disease-free survival; ALN, axillary lymph node; OS, overall survival; HR, hazard ratio; CI, confidence interval.

© 2020 Yu Y et al. *JAMA Network Open.*

eFigure 4. Unsupervised Clustering Analysis of MRI Multisequence Data of Axillary Region of Interest

A. Clustered matrix for multi-sequence profiles of ALN

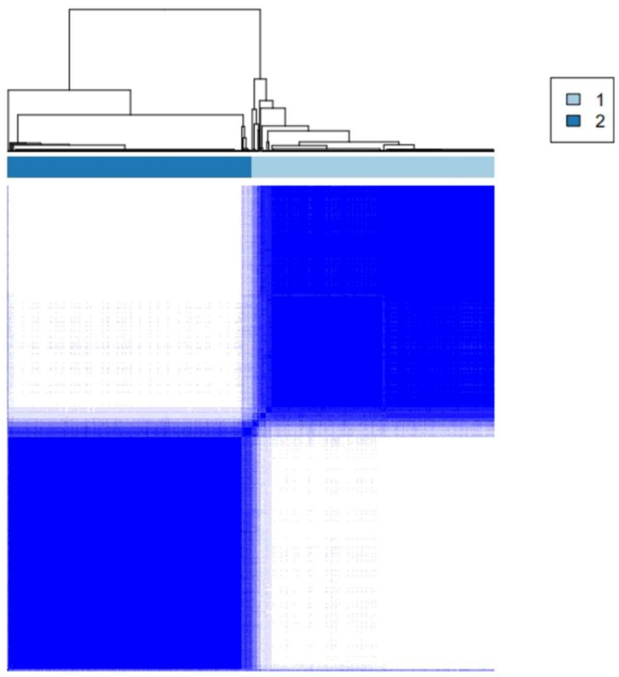

B. Bar chart for cluster of ALN

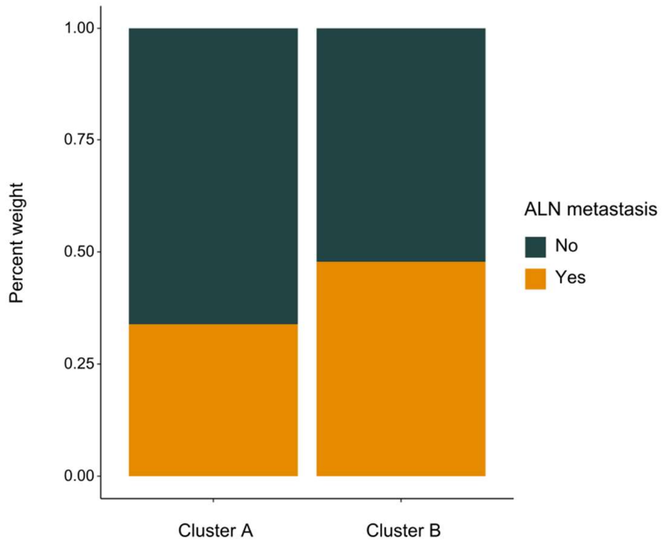

(A) Heatmap illustrating clustered matrix of sample-wise similarities (Entire cohort) based on multi-sequence (T1+C, T2WI and DWI-ADC) radiomic profiles of axillary lymph nodes. (B) Distribution of ALN metastasis over patient clusters defined by similarities of radiomic profile (OR 1.79, 95% CI 1.30-2.46; *P* < .001). MRI, magnetic resonance imaging; ALN, axillary lymph node; T1+C, contrast-enhanced T1-weighted imaging; T2WI, T2-weighted imaging; DWI-ADC, diffusion-weighted imaging quantitatively measured apparent diffusion coefficients; OR, odds ratio; CI, confidence interval.

© 2020 Yu Y et al. *JAMA Network Open*.

eFigure 5. Identification of Essential Radiomic Features of T1+C, T2WI and DWI-ADC Sequences for ALN Metastasis Prediction with LASSO Regression Analysis

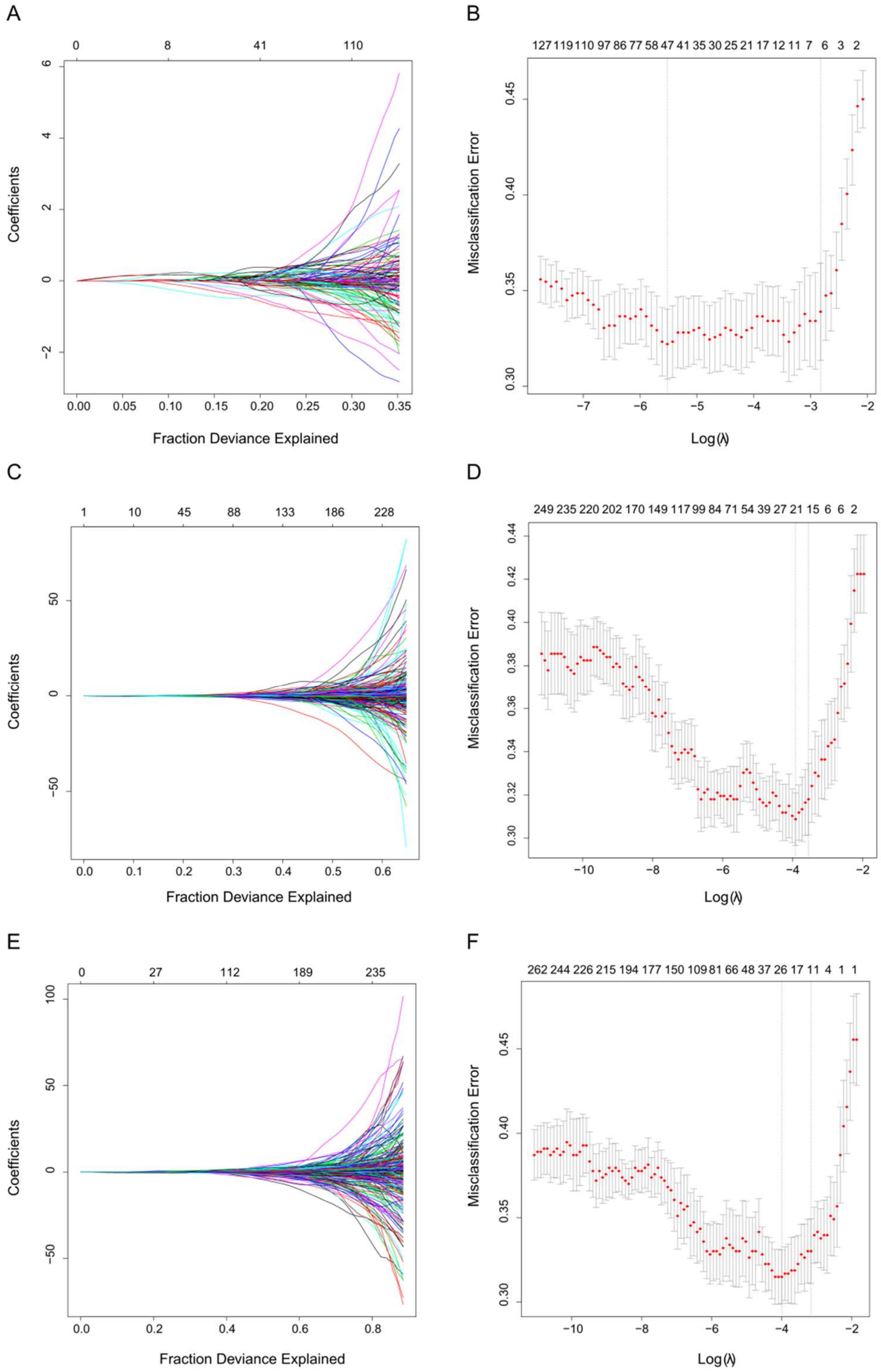

(A-B) T1+C sequence. (C-D) T2WI sequence. (E-F) DWI-ADC sequence. T1+C, contrast-enhanced T1-weighted imaging; T2WI, T2-weighted imaging; DWI-ADC, diffusion-weighted imaging quantitatively measured apparent diffusion coefficients; ALN, axillary lymph node; LASSO, Least absolute shrinkage and selection operator. The LASSO model was used to select 47, 21, and 21 key features from the T1+C, T2WI, and DWI-ADC MRI sequences, respectively.

© 2020 Yu Y et al. *JAMA Network Open*.

eFigure 6. ROC Curves of Radiomic Signature for ALN Metastasis Prediction in the Development and Validation Cohorts Based on LASSO–Logistic Regression Model

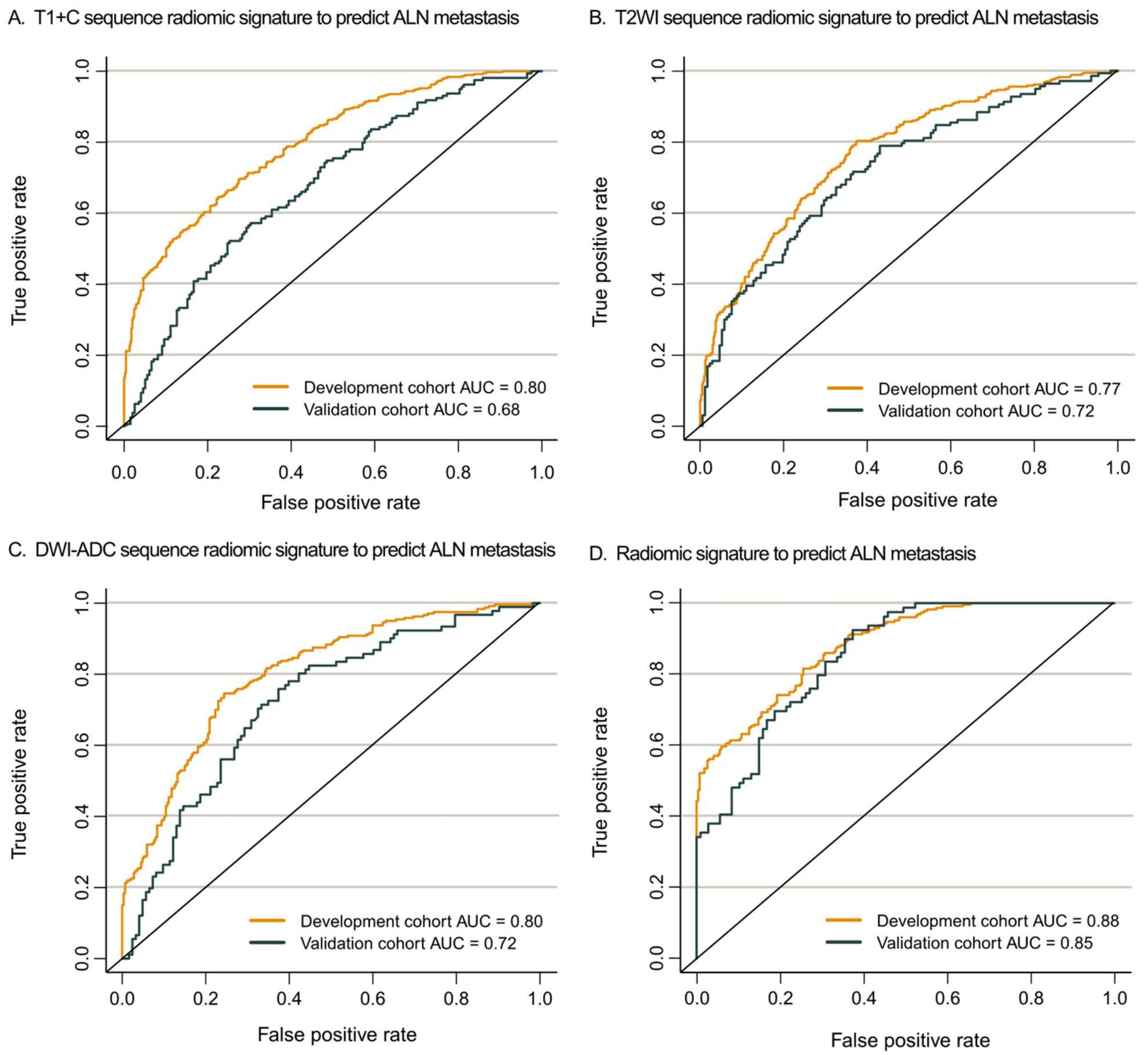

(A) T1+C sequence radiomic signature. (B) T2WI sequence radiomic signature. (C) DWI-ADC sequence radiomic signature. (D) Combination of features from T1+C, T2WI, and DWI-ADC sequences radiomic signature. ROC, receiver operating characteristic; AUC, area under the receiver operating characteristics curve; T1+C, contrast-enhanced T1-weighted imaging; T2WI, T2-weighted imaging; DWI-ADC, diffusion-weighted imaging quantitatively measured apparent diffusion coefficients; ALN, axillary lymph node. The logistic regression model resulted in AUCs of 0.80 and 0.68 for the T1+C signature, 0.77 and 0.72 for the T2WI signature, and 0.80 and 0.72 for the DWI-ADC signature. In addition, this model showed the AUCs of 0.88 and 0.85 for the radiomic signature in the development and validation cohorts, respectively.

© 2020 Yu Y et al. *JAMA Network Open.*

eFigure 7. ROC Curves of Clinical Signature for ALN Metastasis Prediction in the Development and Validation Cohorts

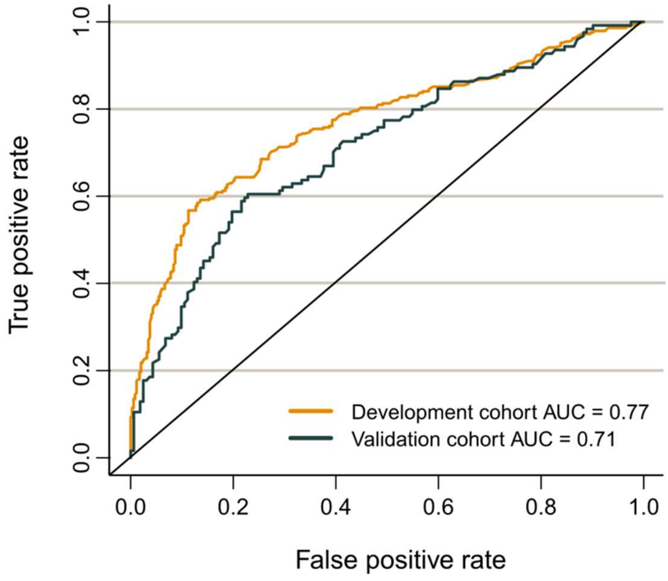

Clinical signature was built based on Logistic regression model. ROC, receiver operating characteristic; AUC, area under the receiver operating characteristics curve; ALN, axillary lymph node.

© 2020 Yu Y et al. *JAMA Network Open.*

eFigure 8. Clinical-Radiomic Nomogram Developed in the Development Cohort to Predict Axillary Lymph Node Metastasis

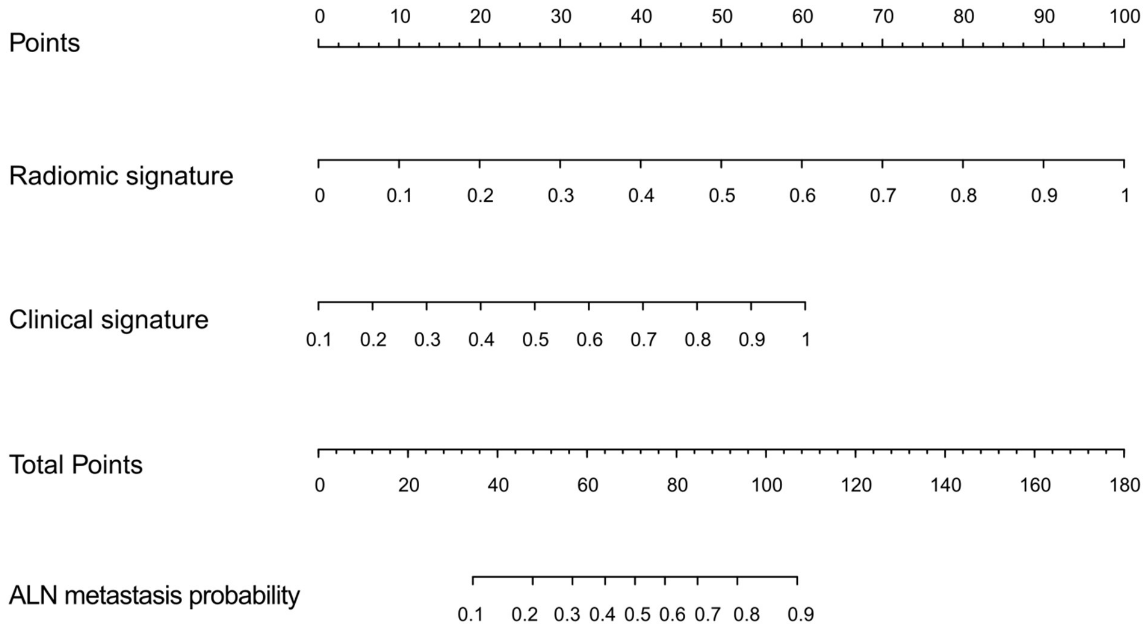

ALN, axillary lymph node.

© 2020 Yu Y et al. *JAMA Network Open*.

eFigure 9. ROC Curves of Clinical-Radiomic Nomogram for ALN Metastasis Prediction in Different Institutions

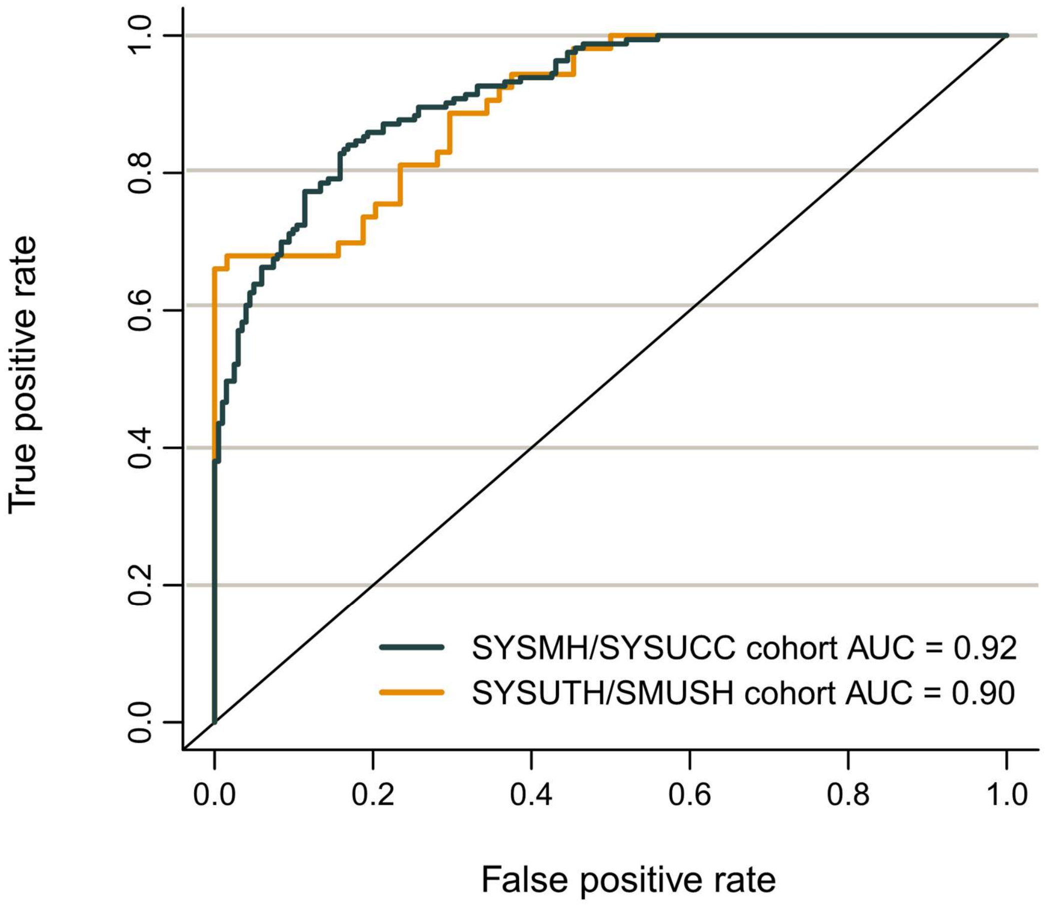

ROC, receiver operating characteristic; AUC, area under the receiver operating characteristics curve; ALN, axillary lymph node

© 2020 Yu Y et al. *JAMA Network Open.*

eFigure 10. ROC Curves of Clinical-Radiomic Nomogram for ALN Metastasis Prediction in Different Molecular Subtype in the Development and Validation Cohorts

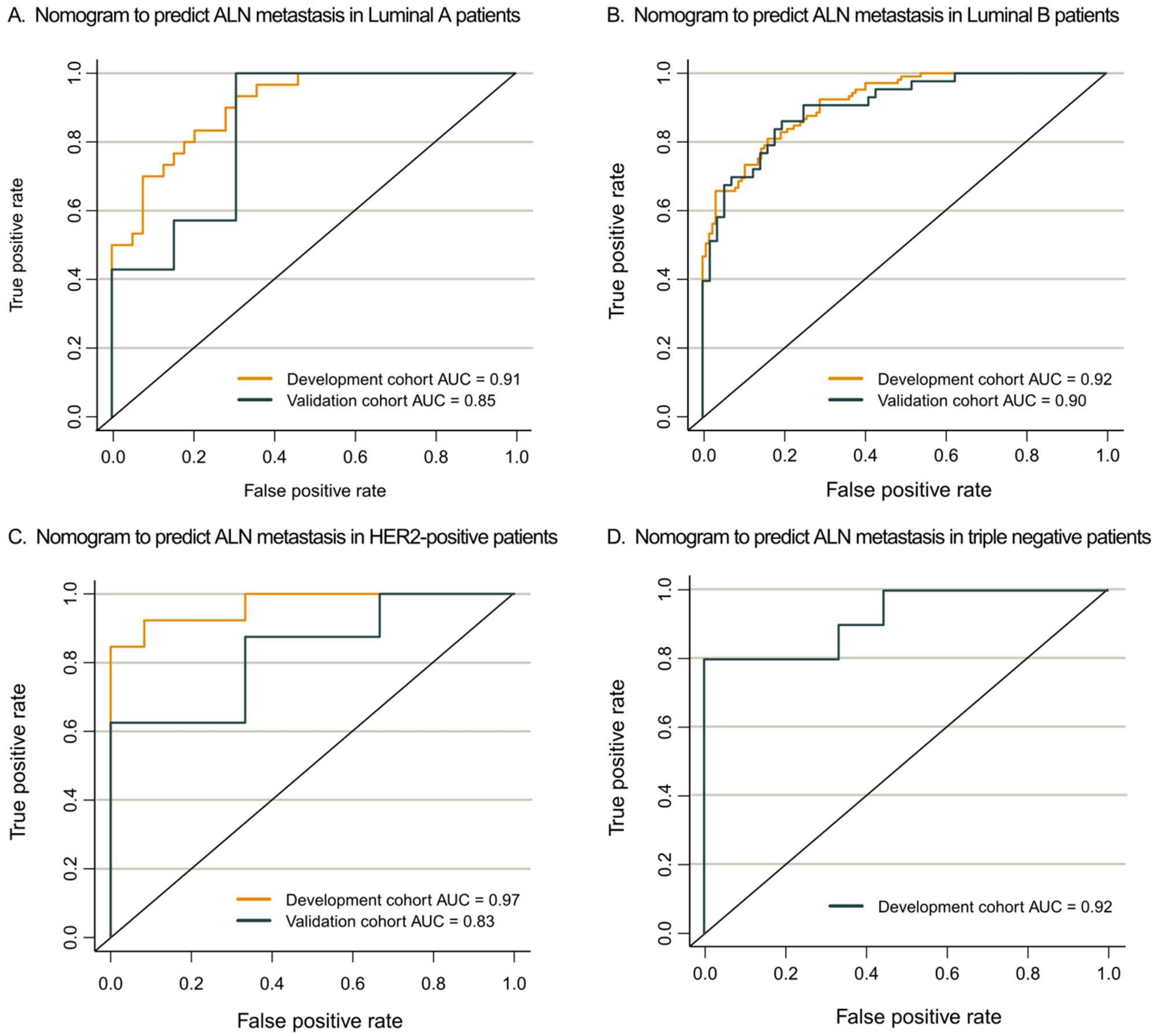

(A) Luminal A. (B) Luminal B. (C) HER2-positive. (D) Triple negative. ROC, receiver operating characteristic; AUC, area under the receiver operating characteristics curve; ALN, axillary lymph node; HER2, human epidermal growth factor receptors 2.

© 2020 Yu Y et al. *JAMA Network Open.*

eFigure 11. Decision Curve Analysis for ALN Metastasis in the Development and Validation Cohorts

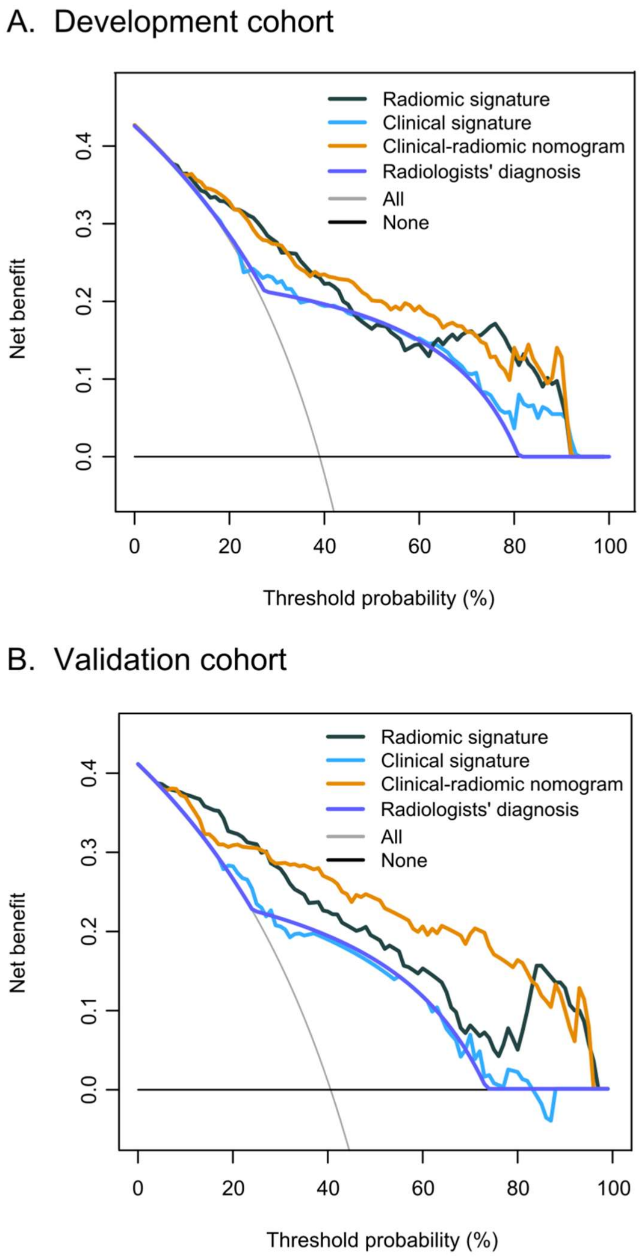

(A) ALN metastasis prediction. (B) DFS prediction. ALN, axillary lymph node; DFS, disease-free survival.

© 2020 Yu Y et al. *JAMA Network Open.*

eFigure 12. Unsupervised Clustering Analysis of MRI Multisequence Data of Primary Breast Tumors

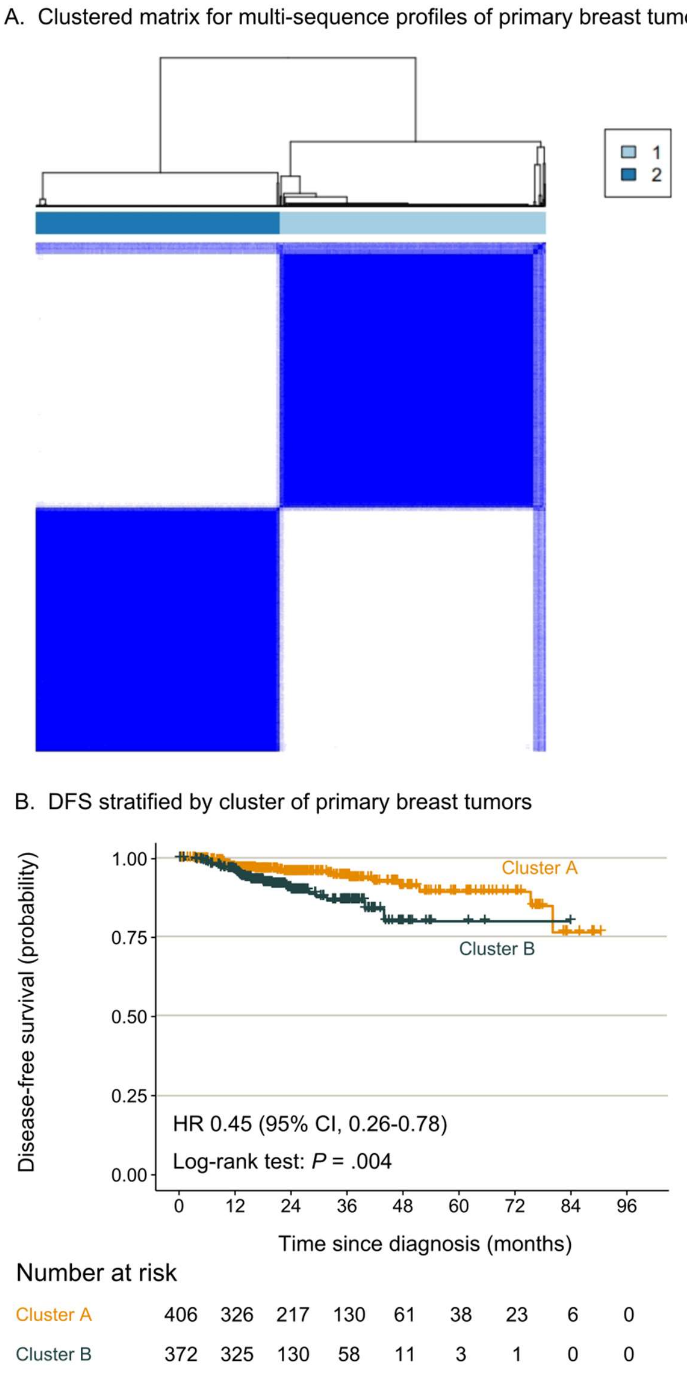

(A) Heatmap illustrating clustered matrix of sample-wise similarities (Entire cohort) based on multi-sequence (T1+C, T2WI and DWI-ADC) radiomic profiles of primary breast tumors. (B) Kaplan−Meier analysis of the MRI radiomic subgroups with DFS. MRI, magnetic resonance imaging; T1+C, contrast-enhanced T1-weighted imaging; T2WI, T2-weighted imaging; DWI-ADC, diffusion-weighted imaging quantitatively measured apparent diffusion coefficients; DFS, disease-free survival; HR, hazard ratio; CI, confidence interval.

© 2020 Yu Y et al. *JAMA Network Open.*

eFigure 13. ROC Curves of T1+C Sequence Radiomic Cignature for 1-Year, 2-Year, and 3-Year DFS Prediction based on the Random forest-Cox Regression Model

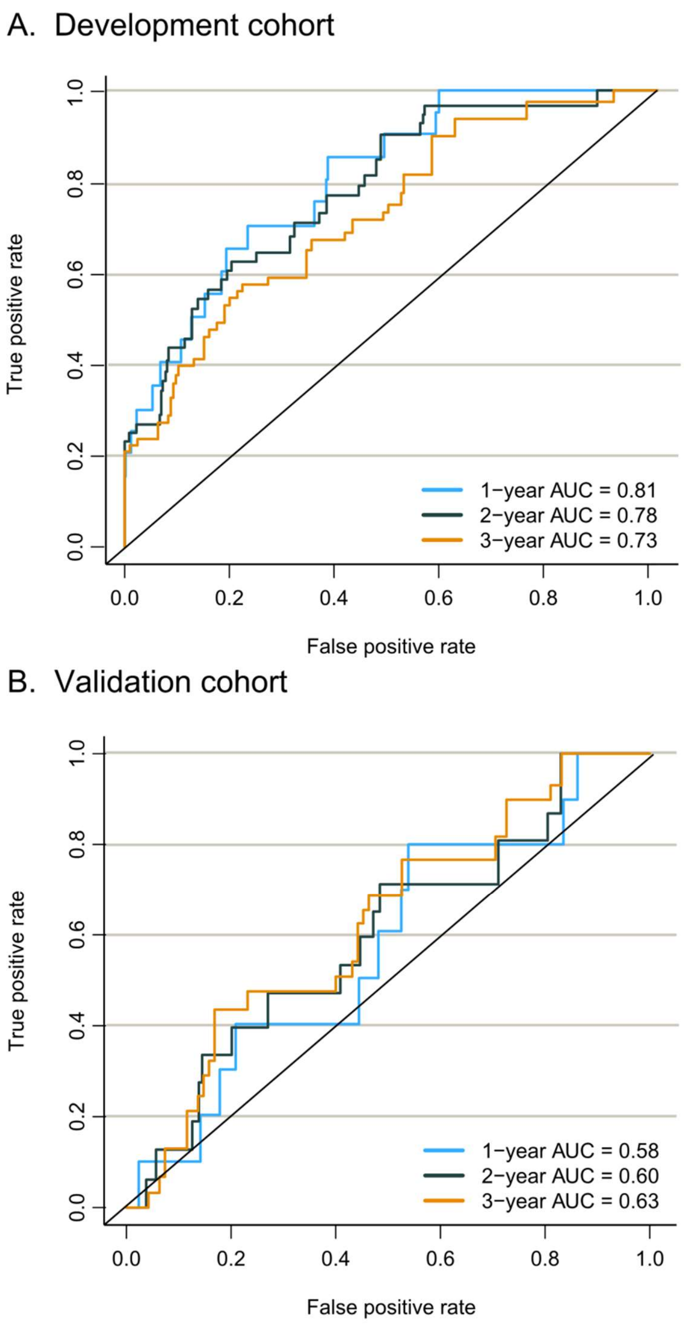

(A) Development cohort. (B) Validation cohort. T1+C, contrast-enhanced T1-weighted imaging; DFS, disease-free survival; ROC, receiver operating characteristic; AUC, area under the receiver operating characteristics curve. The top 30 features identified by the random forest algorithm were also selected and used to construct a model with the coefficients weighted using the penalized Cox model that showed AUCs for the 1-, 2-, and 3-year DFS of 0.81, 0.78, and 0.73, respectively in the development cohort, for T1+C signature; and also predicted AUCs of 0.58, 0.60, and 0.63 in the validation cohort, respectively.

© 2020 Yu Y et al. *JAMA Network Open.*

eFigure 14. ROC Curves of T2WI Sequence Radiomic Signature for 1-Year, 2-Year, and 3-Year DFS Prediction based on the Random Forest–Cox Regression Model

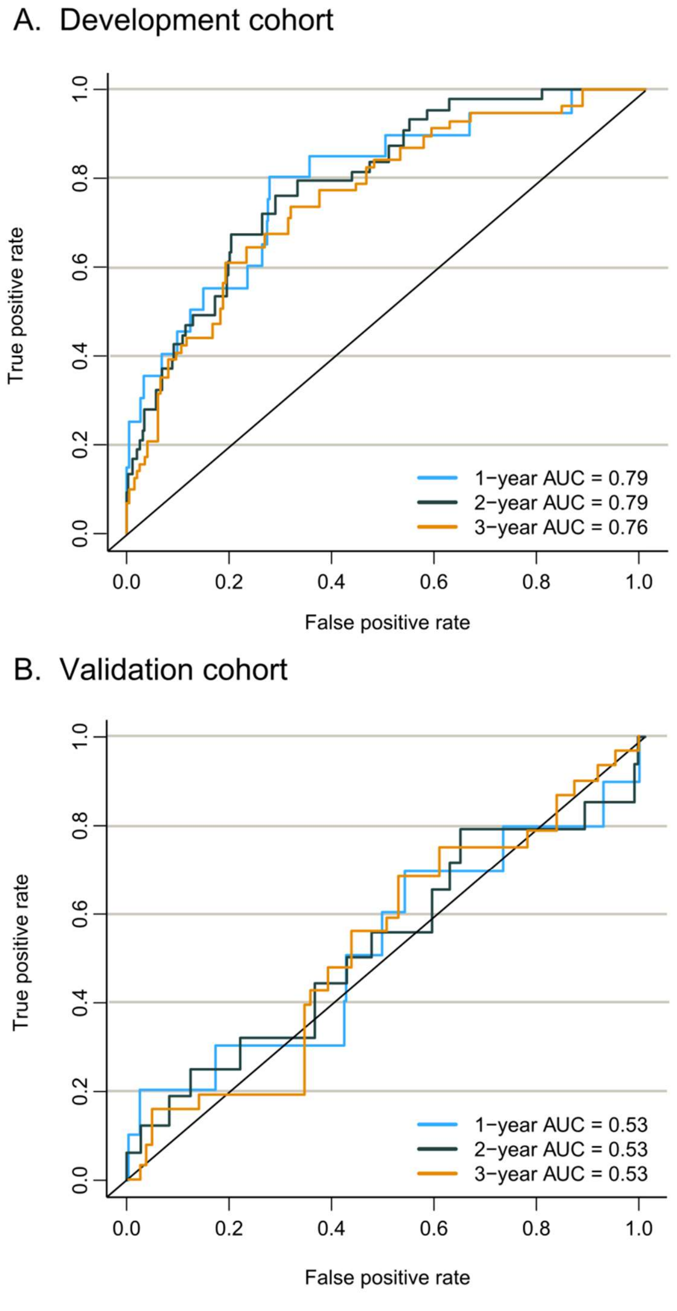

(A) Development cohort. (B) Validation cohort. T2WI, T2-weighted imaging; DFS, disease-free survival; ROC, receiver operating characteristic; AUC, area under the receiver operating characteristics curve. The top 30 features identified by the random forest algorithm were also selected and used to construct a model with the coefficients weighted using the penalized Cox model that showed AUCs for the 1-, 2-, and 3-year DFS of 0.79, 0.79, and 0.76 for the T2WI signature in the development cohort, respectively; also predicted AUCs of 0.53, 0.53, and 0.53 in the validation cohort, respectively.

© 2020 Yu Y et al. *JAMA Network Open.*

eFigure 15. ROC curves of DWI-ADC Sequence Radiomic Signature for 1-Year, 2-Year, and 3-Year DFS Prediction based on the Random Forest–Cox Regression Model

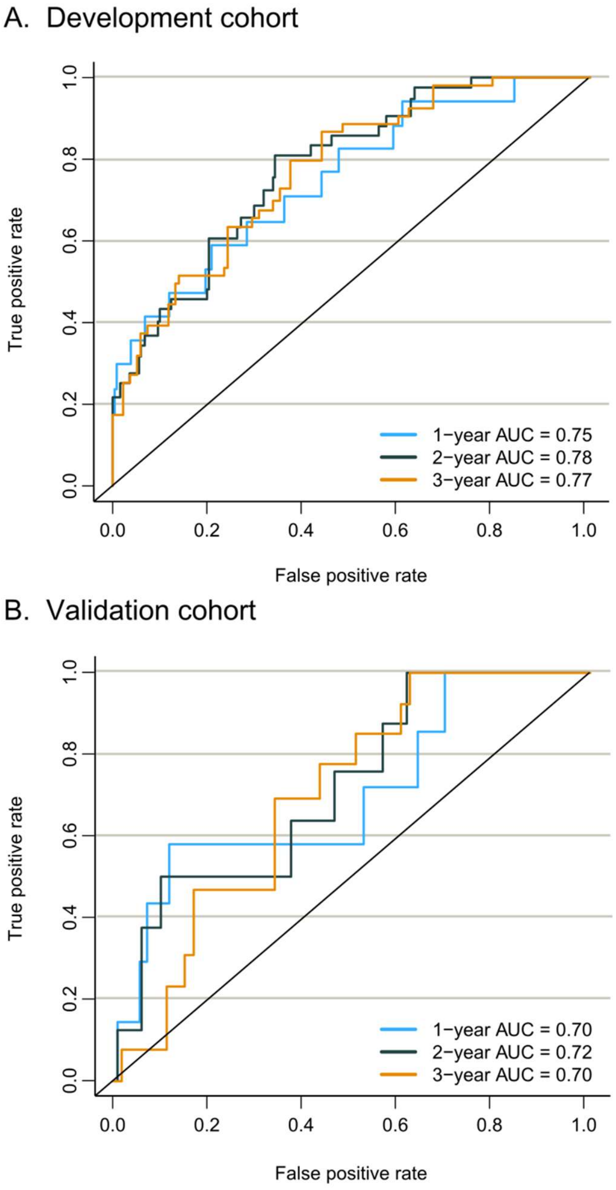

(A) Development cohort. (B) Validation cohort. DWI-ADC, diffusion-weighted imaging quantitatively measured apparent diffusion coefficients; DFS, disease-free survival; ROC, receiver operating characteristic; AUC, area under the receiver operating characteristics curve. The top 30 features identified by the random forest algorithm were also selected and used to construct a model with the coefficients weighted using the penalized Cox model that showed AUCs for the 1-, 2-, and 3-year DFS of 0.75, 0.78, and 0.77 for the DWI-ADC signature in the development cohort, respectively; also predicted AUCs of 0.70, 0.72, and 0.70 in the validation cohort, respectively.

© 2020 Yu Y et al. *JAMA Network Open*.

eFigure 16. The Performance of Radiomic Signature Combining T1+C, T2WI, and DWI-ADC Features in Predicting DFS based on the Random Forest–Cox Regression Model

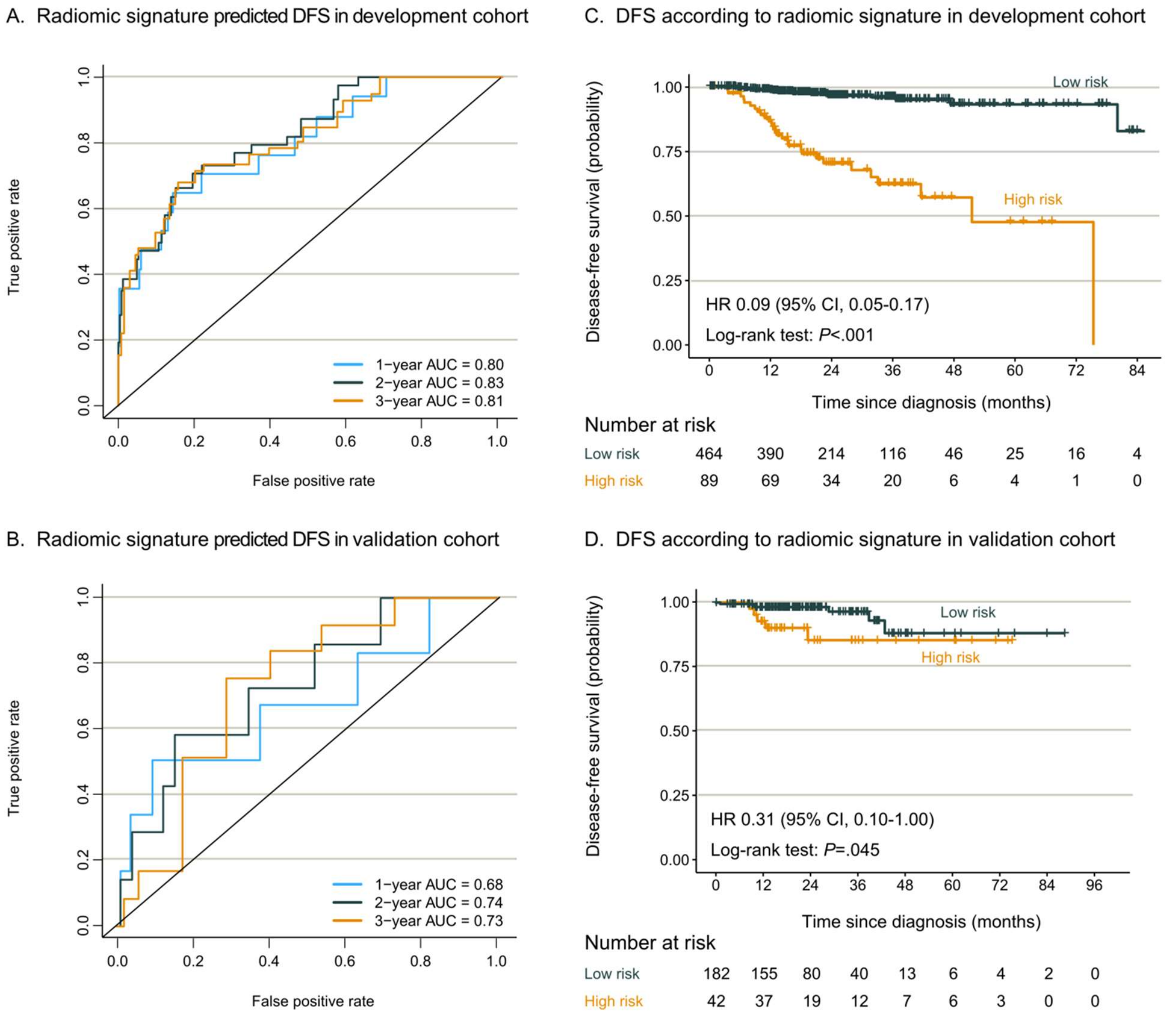

ROC curves of radiomic signature for 1, 2 and 3-year DFS prediction in the development (A) and validation (B) cohorts, respectively. Kaplan-Meier plots for DFS according to radiomic signature in the development (C) and validation (D) cohorts, respectively. DFS, disease-free survival; HR, hazard ratio; CI, confidence interval; ROC, receiver operating characteristic; AUC, area under the receiver operating characteristics curve.

© 2020 Yu Y et al. *JAMA Network Open.*

eFigure 17. Univariate Association of Clinicopathological Characteristics with DFS

	Events (n)/ patients (N) in patients with risk factor	Events (n)/ patients (N) in patients without risk factor	HR	(95%CI)	*P* value
**Age (years)(≥40 vs ＜40)**					
Development cohort	44/702	16/147	0.59	(0.33-1.05)	.070
Validation cohort	18/286	8/79	0.66	(0.29-1.51)	.320
**Number of tumors (＞1 vs 1)**					
Development cohort	15/123	45/725	2.12	(1.18-3.80)	.010
Validation cohort	5/47	21/318	1.90	(0.71-5.04)	.190
**Histological type (Others vs Invasive ductal carcinoma)**					
Development cohort	4/91	56/758	0.52	(0.19-1.44)	.200
Validation cohort	1/33	25/332	0.38	(0.05-2.82)	.320
**Histological grade (Grade3 (high) vs Grade1-2 (low-intermediate))**					
Development cohort	31/328	19/383	2.00	(1.13-3.55)	.015
Validation cohort	16/139	6/183	3.73	(1.46-9.56)	.003
**Pathological T stage (T2-4 vs T1)**					
Development cohort	36/435	24/408	1.44	(0.86-2.41)	.170
Validation cohort	18/176	7/188	2.78	(1.16-6.65)	.017
**Pathological N stage (N1-3 vs N0)**					
Development cohort	41/382	19/467	2.55	(1.48-4.40)	<.001
Validation cohort	19/162	7/203	3.75	(1.57-8.92)	.001
**Pathological TNM stage (II-III vs I)**					
Development cohort	48/579	12/259	1.74	(0.92-3.28)	.083
Validation cohort	22/249	3/113	3.54	(1.06-11.84)	.028
**ER status (Positive vs Negative)**					
Development cohort	51/714	9/133	1.05	(0.52-2.14)	.890
Validation cohort	19/302	7/60	0.52	(0.22-1.25)	.140
**PR status (Positive vs Negative)**					
Development cohort	34/608	26/238	0.49	(0.30-0.82)	.005
Validation cohort	15/251	11/111	0.57	(0.26-1.25)	.160
**HER2 status (Positive vs Negative)**					
Development cohort	19/224	31/524	1.27	(0.72-2.25)	.410
Validation cohort	13/114	12/206	2.19	(1.00-4.82)	.044
**Ki67 status (≥30 vs ＜30)**					
Development cohort	34/379	25/464	1.98	(1.18-3.34)	.009
Validation cohort	14/159	11/202	2.26	(1.02-5.02)	.040
**Molecular subtype (TN/HER2+ vs Luminal)**					
Development cohort	9/121	51/721	1.10	(0.54-2.23)	.800
Validation cohort	6/55	19/306	1.79	(0.72-4.49)	.210
**Breast conversing surgery (Yes vs No)**					
Development cohort	15/343	45/505	0.49	(0.27-0.87)	.014
Validation cohort	8/163	18/202	0.54	(0.23-1.24)	.140

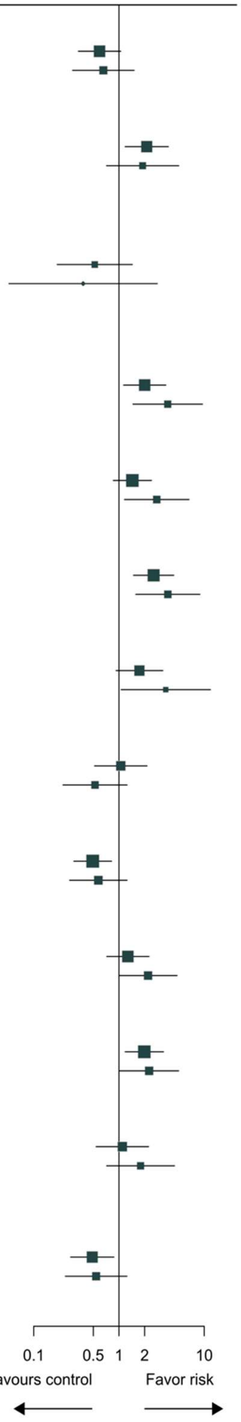

DFS, disease-free survival; TNM, tumor–node–metastasis; HER2, human epidermal growth factor receptors 2; ER, estrogen receptor; PR, progesterone receptors; TN, triple negative; HER2, human epidermal growth factor receptors 2; HR, hazard ratio; CI, confidence interval.

© 2020 Yu Y et al. *JAMA Network Open*.

eFigure 18. The Performance of Clinical Signature in Predicting DFS

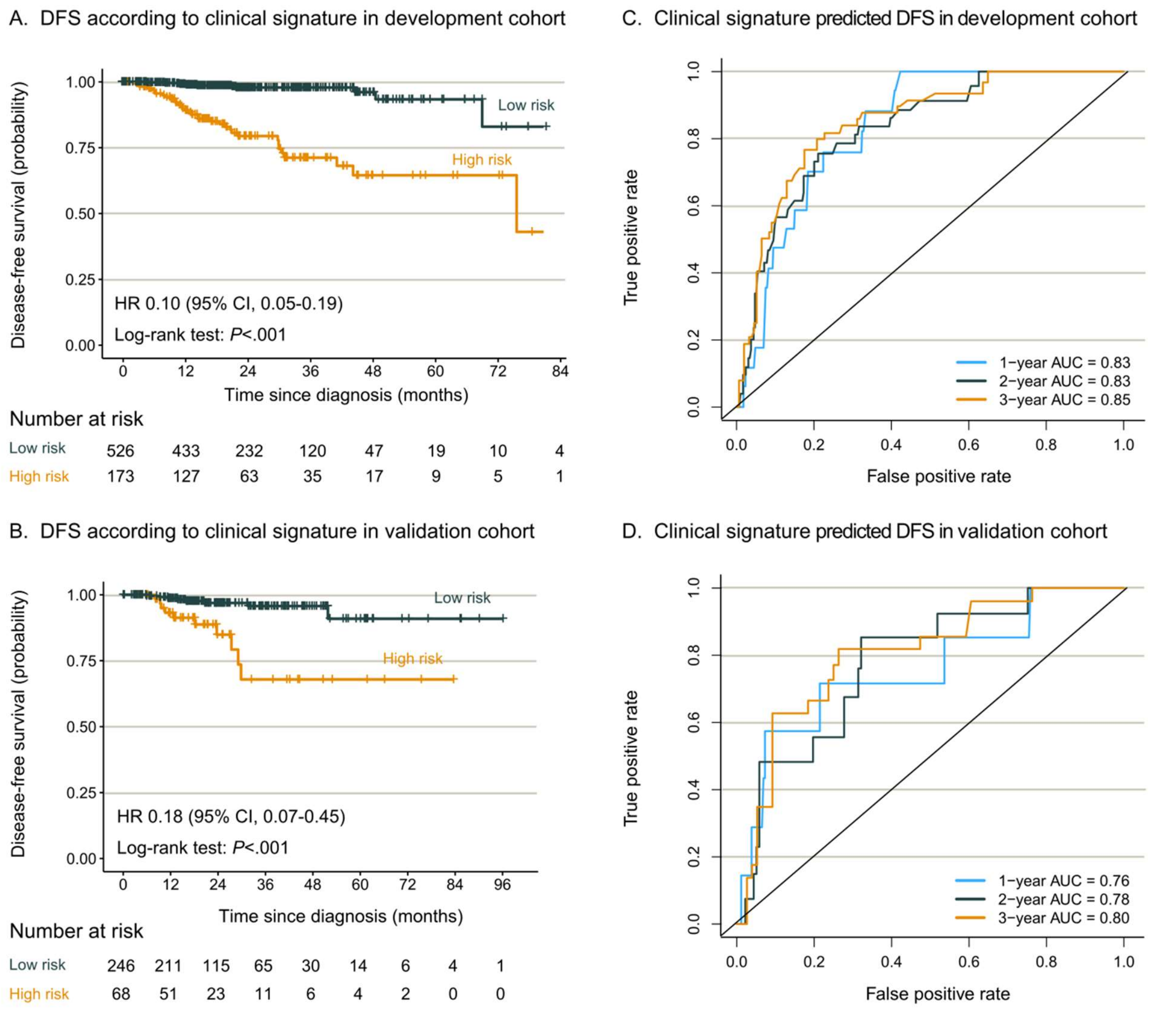

Kaplan-Meier plots for DFS according to clinical signature in the development (A) and validation (B) cohorts, respectively. ROC curves of clinical signature for 1, 2 and 3-year DFS prediction in the development (C) and validation (D) cohorts, respectively. DFS, disease-free survival; HR, hazard ratio; CI, confidence interval; ROC, receiver operating characteristic; AUC, area under the receiver operating characteristics curve.

© 2020 Yu Y et al. *JAMA Network Open*.

eFigure 19. Clinical-Radiomic Nomogram Developed in the Development Cohort to Predict 1-Year, 2-Year, and 3-Year DFS

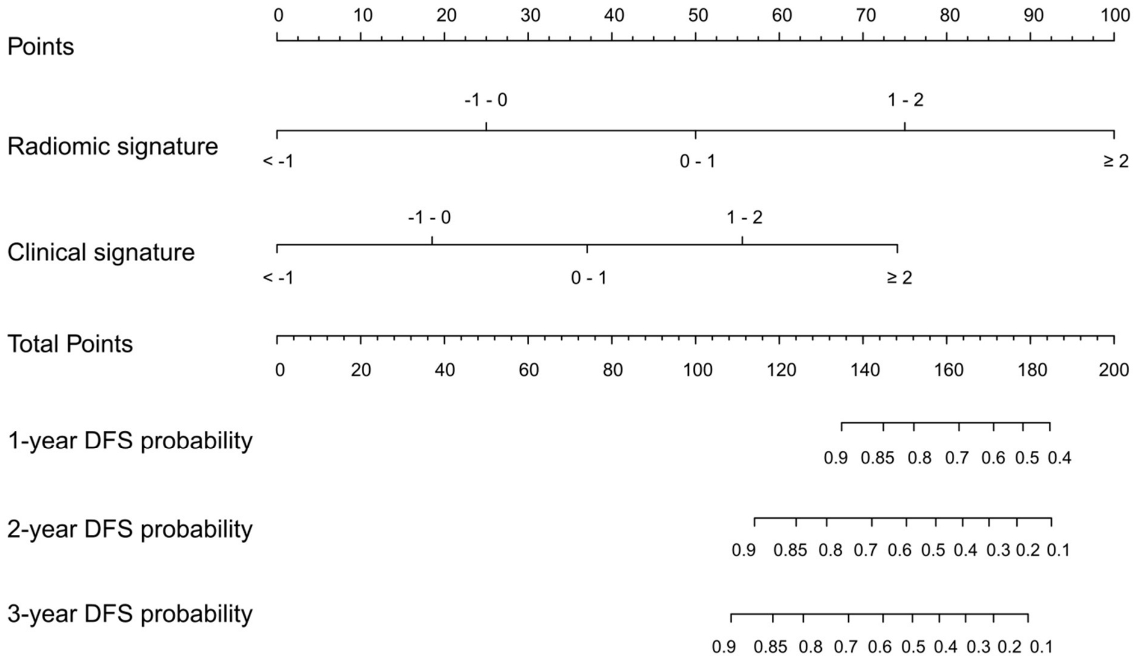

DFS, disease-free survival.

© 2020 Yu Y et al. *JAMA Network Open*.

eFigure 20. ROC Curves of Clinical-radiomic Nomogram for 1-Year, 2-Year, and 3-year DFS Prediction in Different Institutions

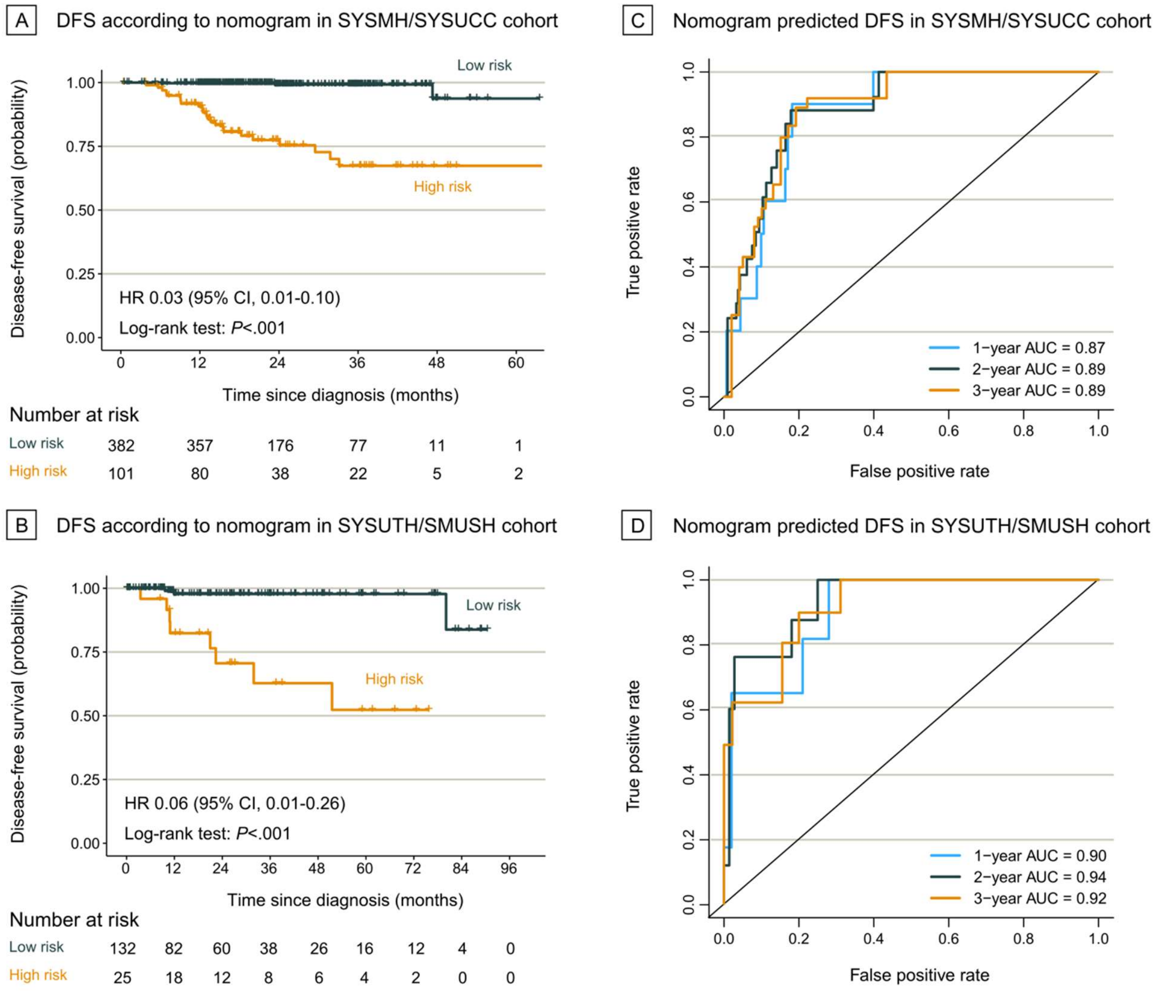

Kaplan-Meier plots for DFS according to clinical-radiomic nomogram in the SYSMH/SYSUCC (A) and SYSUTH/SMUSH (B) cohorts, respectively. ROC curves of clinical-radiomic nomogram for 1, 2 and 3-year DFS prediction in the SYSMH/SYSUCC (A) and SYSUTH/SMUSH (B) cohorts, respectively. DFS, disease-free survival; HR, hazard ratio; CI, confidence interval; ROC, receiver operating characteristic; AUC, area under the receiver operating characteristics curve.

© 2020 Yu Y et al. *JAMA Network Open.*

eFigure 21. ROC Curves of Clinical-Radiomic Nomogram for 1-Year, 2-Year, and 3-year DFS Prediction in Different Molecular Subtypes in the Entire Cohort

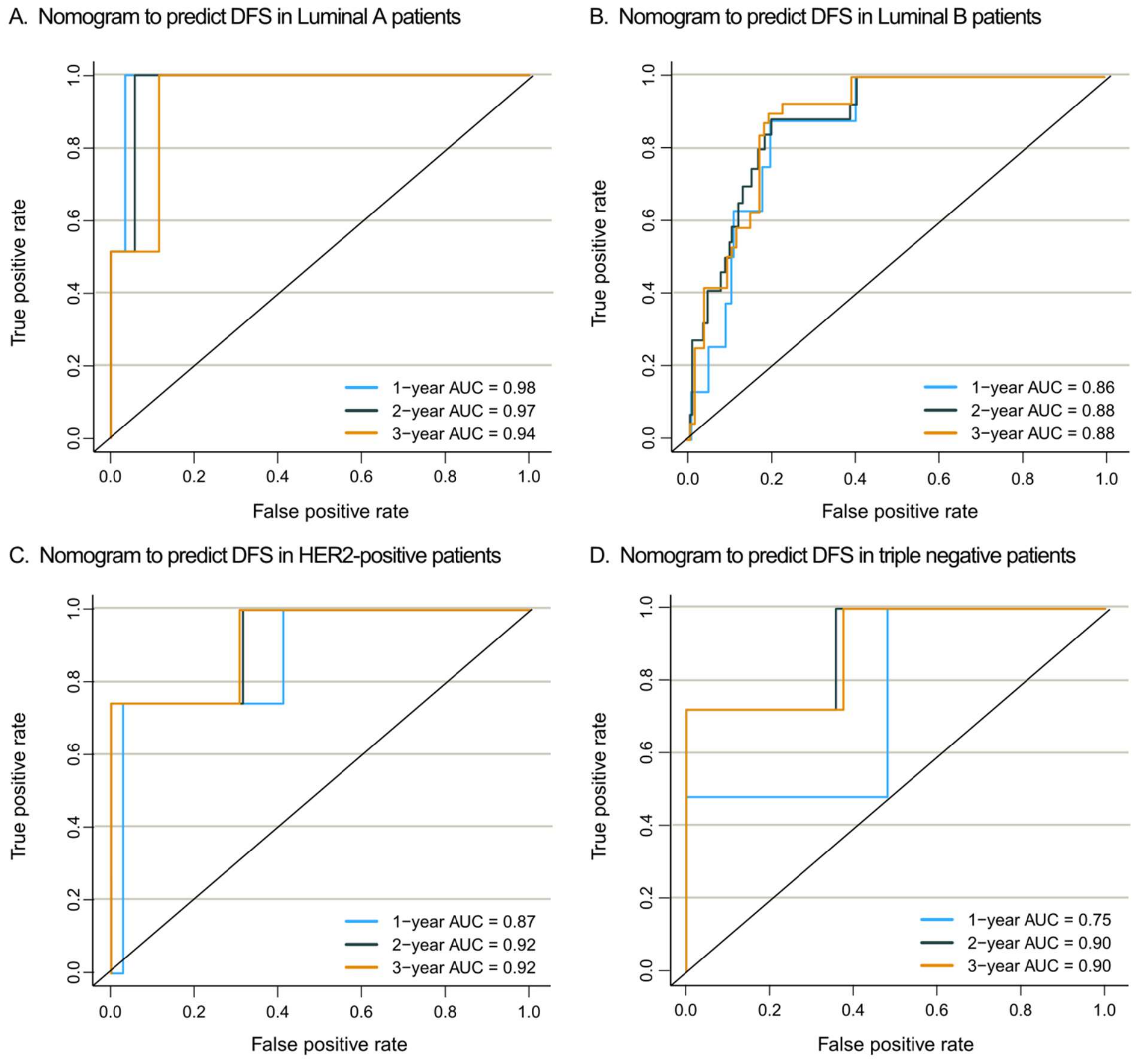

(A) Luminal A. (B) Luminal B. (C) HER2-positive. (D) Triple negative. DFS, disease-free survival; ROC, receiver operating characteristic; AUC, area under the receiver operating characteristics curve; HER2, human epidermal growth factor receptors 2.

© 2020 Yu Y et al. *JAMA Network Open.*

eFigure 22. Decision Curve Analysis for DFS Prediction in the Development and Validation Cohorts

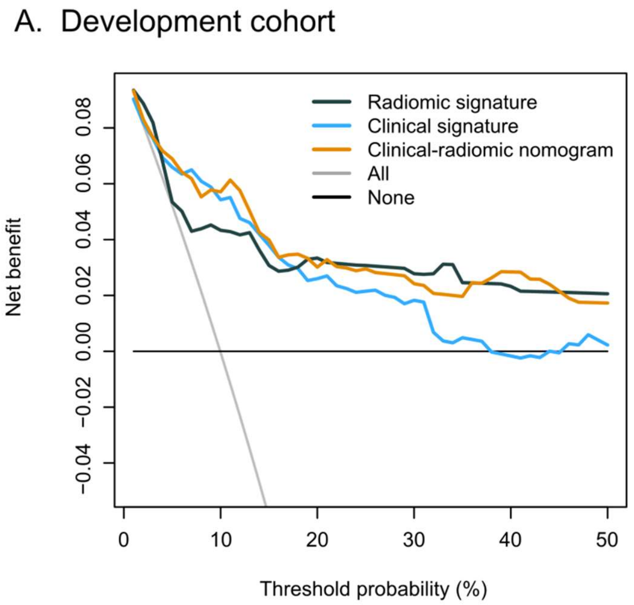

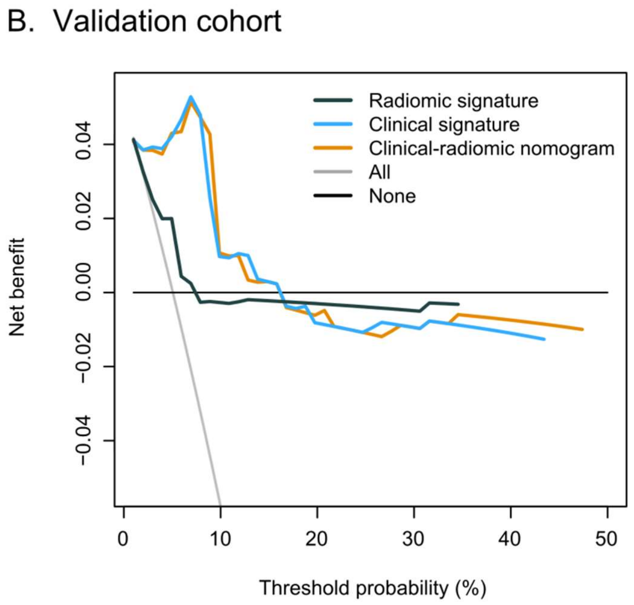

(A) Development cohort. (B) Validation cohort. DFS, disease-free survival.

© 2020 Yu Y et al. *JAMA Network Open.*

eTable 1. Information of 4 Institutions in This Study

Institution	Investigator in Charge	No. of Patients Enrolled	Time of Patients Enrolled
Sun Yat-Sen Memorial Hospital	Herui Yao	622	November 17, 2011 to December 25, 2018
Sun Yat-Sen University Cancer Center	Chuanmiao Xie	381	July 03, 2007 to August 26, 2019
Tungwah Hospital of Sun Yat-Sen University	Jie Ouyang	90	July 08, 2013 to September 21, 2019
Shunde Hospital of Southern Medical University	Qiugen Hu	121	March 09, 2012 to December 18, 2018

© 2020 Yu Y et al. *JAMA Network Open.*

eTable 2. MR Scanning Parameters for the Patients

Hospital	Scanner	Sequence	TR/TE (ms)	FOV (mm)	Matrix	Slice Thickness (mm)	Slice Gap (mm)	Slices	Flip Angle	Acquisition Time (min)	Scans
**SYSMH**	Philips 1.5T (Achieva)	T2WI	4000/60	337×240	400×318	3	0	55	90°	4min	
		DWI-ADC	7439/53	363×340	360×301	3	0	55	90°	4min35s	
		T1+C	3.3/1.54	320×250	217×172	1	0	55	10°	7min	55
	Philips 3.0T (Ingenia)	T2WI	4000/60	337×240	400×318	3	0	55	90°	4min	
		DWI-ADC	7439/53	363×340	360×301	3	0	55	90°	4min35s	
		T1+C	3.3/1.54	320×250	217×172	1	0	55	10°	7min	55
	Siemens 1.5T (Avanto)	T2WI	2760/107	350×350	320×224	5	1	30	150°	2min46s	
		DWI-ADC	5400/119	400×252	200×170	6	1.8	20	180°	2min34s	
		T1+C	4.95/2.2	380×269	288×216	3	0.6	48-72	10°	5-7min	50/70
	Siemens 3.0T (Skyra)	T2WI	7600/75	340×340	448×358	4	0.8	35	116°	3min42s	
		DWI-ADC	7620/64	360×310	192×192	4	0.8	35	180°	1min54s	
		T1+C	3.25/1.22	380×327	256×218	2.5	0.5	48-72	10°	5-7min	50/70

© 2020 Yu Y et al. *JAMA Network Open.*

**SYSUCC**	United Imaging 3.0T (China)	T2WI	3600/74.34	340×340	336×335	5	1	24	90°	3min05s	
		DWI-ADC	3597/67.2	350×350	350×190	6	1	24	90°	1min33s	
		T1+C	4.3/1.99	340×340	336×335	0.67	0	204	10°	9min58s	8
	GE 3.0T (USA)	T2WI	3912/107.64	380×380	416×256	5	1	28	111°	1min59s	
		DWI-ADC	4168/60.2	380×380	128×160	5	1	48	-	1min40s	
		T1+C	4.3/1.7	360×360	256×320	1	0	204	5°	9min50s	8

© 2020 Yu Y et al. *JAMA Network Open.*

Hospital	Scanner	Sequence	TR/TE (ms)	FOV (mm)	Matrix	Slice Thickness (mm)	Slice Gap (mm)	Slices	Flip Angle	Acquisition Time (min)	Scans
**SYSUTH**	Philips 1.5T (Achieva)	T2WI	3400/90	260×320	348×299	3	0.3	44	120°	4min4s	
		DWI-ADC	2000/103	320×320	160×160	5	1	32	90°	1min36s	
		T1+C	5.4/2.4	300×320	300×320	1	0	300	15°	7min2s	6
	Philips 3.0T (Ingenia)	T2WI	4495/70	280×340	332×377	3	0	52	90°	3min53s	
		DWI-ADC	7011/67	320×340	148×153	4	1	30	90°	1min24s	
		T1+C	4.8/2.1	280×340	280×339	1	0	300	12°	6min59s	6
**SMUSH**	Philips 1.5T (Achieva)	T2WI	4518/70	320×260	256×159	4	0.07	36	120°	3min19s	
		DWI-ADC	5837/67	365×221	124×73	3	1	36	90°	3min59s	
		T1+C	6.0/2.9	350×255	252×243	2	1	150	10°	6min42s	9
	Siemens 3.0T (Skyra)	T2WI	4290/78	128×332	332×128	4	1	30	180°	1min22s	
		DWI-ADC	6560/49	340×172	66×130	4	0.8	37	180°	3min51s	
		T1+C	5.59/1.96	154×338	562×256	2	0.4	72	10°	7min15s	6

Abbreviations: FOV, field of view; TR, repetition time; TE, echo time; T1+C, contrast-enhanced T1-weighted imaging; T2WI, T2-weighted imaging; DWI-ADC, diffusion-weighted imaging quantitatively measured

© 2020 Yu Y et al. *JAMA Network Open.*

apparent diffusion coefficients; SYSMH, Sun Yat-sen Memorial Hospital of Sun Yat-sen University; SYSUCC, Sun Yat-sen University Cancer Center; SYSUTH, Tungwah Hospital of Sun Yat-sen University; SMUSH, Shunde Hospital of Southern Medical University.

© 2020 Yu Y et al. *JAMA Network Open.*

eTable 3. The Performance to Predict Axillary Lymph Node Metastasis Among Signatures Constructed by Different Algorithms

		Feature Selection		LASSO			Random Forest	
**Signature**	**Cohort**	**Classifer**		**Logistic Regression**	**SVM**		**Logistic Regression**	**SVM**
				**(AUC)**	**(AUC)**		**(AUC)**	**(AUC)**
T1+C sequence radiomic signature								
	Development			0.80	0.81		0.77	0.77
	Validation			0.68	0.68		0.54	0.65
T2WI sequence radiomic signature								
	Development			0.77	0.82		0.79	0.79
	Validation			0.72	0.69		0.62	0.68
DWI-ADC sequence radiomic signature								
	Development			0.80	0.82		0.79	0.79
	Validation			0.72	0.74		0.71	0.75
Radiomic signature								
	Development			0.88	0.86		0.94	0.86

© 2020 Yu Y et al. *JAMA Network Open.*

	Validation			0.85	0.74		0.53	0.75
Abbreviations: LASSO, Least absolute shrinkage and selection operator; SVM, support vector machine; AUC, area under the receiver operating characteristics curve; T1+C, contrast-enhanced T1-weighted imaging; T2WI, T2-weighted imaging; DWI-ADC, diffusion-weighted imaging quantitatively measured apparent diffusion coefficients.								

© 2020 Yu Y et al. *JAMA Network Open.*

eTable 4. The Performance of Different Region of Interest Constructed for Axillary Lymph Node Metastasis Prediction Based on LASSO–Logistic Regression Model

**Signature Performance**	**ALN radiomic signature**			**Tumor radiomic signature**			**Combination of tumor and ALN radiomic signature**	
	Training Cohort	Validation Cohort		Training Cohort	Validation Cohort		Training Cohort	Validation Cohort
**AUC**	0.88	0.85		0.86	0.60		0.90	0.71

Abbreviations: AUC, area under the receiver operating characteristics curve; ALN, axillary lymph node.

© 2020 Yu Y et al. *JAMA Network Open.*

**eTable 5.** Essential Radiomic Features and Formula Composition for ALN Metastasis Prediction based on LASSO–Logistic Regression Model		
**Model**	**Intercept/Feature Name**	**Coefficient**
**T1+C sequence signature**	Intercept=0.0117	β
	wavelet-HHL-glrlm-LongRunEmphasis	0.8756
	wavelet-LLL-glrlm-RunPercentage	-0.5771
	wavelet-HHH-glrlm-RunPercentage	-1.1337
	wavelet-LLH-gldm-LargeDependenceEmphasis	0.3085
	wavelet-LHL-gldm-LargeDependenceEmphasis	-0.6641

© 2020 Yu Y et al. *JAMA Network Open.*

	wavelet-HHH-gldm-LargeDependenceLowGrayLevelEmphasis	0.2296
	wavelet-LHH-gldm-LargeDependenceLowGrayLevelEmphasis	0.3373
	wavelet-HLH-glszm-LargeAreaHighGrayLevelEmphasis	0.2097
	wavelet-HHH-glszm-LargeAreaHighGrayLevelEmphasis	-0.4906
**Model**	**Intercept/Feature Name**	**Coefficient**
	wavelet-LHH-glrlm-RunLengthNonUniformity	-0.6072
	wavelet-LHL-glrlm-GrayLevelNonUniformity	0.2640
	wavelet-LHL-glrlm-LongRunLowGrayLevelEmphasis	0.5809

© 2020 Yu Y et al. *JAMA Network Open.*

	wavelet-HHH-glszm-ZoneVariance	0.9074
	original-gldm-LargeDependenceHighGrayLevelEmphasis	0.3005
	wavelet-LLL-glszm-LargeAreaEmphasis	1.3307
	wavelet-LHH-ngtdm-Coarseness	0.7035
	wavelet-LHL-gldm-DependenceNonUniformity	0.3525
	wavelet-HHH-glszm-LargeAreaEmphasis	-0.1849
	wavelet-HLL-glrlm-RunPercentage	0.3038
	wavelet-LLH-glrlm-RunLengthNonUniformity	0.5444
	wavelet-HLH-glszm-LargeAreaLowGrayLevelEmphasis	0.6363
	wavelet-HHL-glcm-MCC	0.3582

© 2020 Yu Y et al. *JAMA Network Open.*

Model	Intercept/Feature Name	Coefficient
	wavelet-LHH-glrlm-RunPercentage	0.7261
	wavelet-LLH-glrlm-RunVariance	0.2819
	wavelet-LLH-gldm-LargeDependenceHighGrayLevelEmphasis	0.2947
	original-glrlm-LongRunLowGrayLevelEmphasis	-1.0968
	wavelet-LHL-glcm-Idn	-0.4321
	wavelet-LHL-gldm-LargeDependenceLowGrayLevelEmphasis	-0.0675
	original-firstorder-Maximum	0.0539

© 2020 Yu Y et al. *JAMA Network Open.*

	wavelet-HLL-glrlm-ShortRunEmphasis	0.2819
	wavelet-LLL-glrlm-RunVariance	0.6957
	wavelet-LHH-gldm-SmallDependenceEmphasis	-1.1549
	wavelet-LHL-gldm-LargeDependenceHighGrayLevelEmphasis	-0.0573
	wavelet-HLL-gldm-LargeDependenceHighGrayLevelEmphasis	-0.1610
	wavelet-LLH-glrlm-RunEntropy	0.5479
**Model**	**Intercept/Feature Name**	**Coefficient**
	wavelet-LLH-gldm-LargeDependenceLowGrayLevelEmphasis	-0.1522

© 2020 Yu Y et al. *JAMA Network Open.*

	wavelet-LLL-glcm-InverseVariance	1.0442
	wavelet-LHL-glcm-Imc2	0.0455
	wavelet-LLL-glcm-MCC	-0.1096
	wavelet-LLH-ngtdm-Coarseness	-0.1286
	wavelet-LLL-glcm-ClusterShade	-0.1054
	wavelet-HHL-glrlm-RunLengthNonUniformityNormalized	0.7591
	wavelet-HLH-gldm-SmallDependenceEmphasis	-0.3403
	original-shape-MajorAxisLength	-0.5406
	wavelet-LHH-glcm-Imc2	0.3851
	wavelet-LHH-gldm-LargeDependenceHighGrayLevelEmphasis	-0.3265

© 2020 Yu Y et al. *JAMA Network Open.*

	wavelet-HHL-gldm-DependenceVariance	-0.6298
**Model**	**Intercept/Feature Name**	**Coefficient**
**T2WI sequence signature**	Intercept=18.5770	β
	wavelet-HHH-glrlm-RunPercentage	0.0642
	wavelet-HHL-glrlm-LongRunEmphasis	0.1320
	original-gldm-LargeDependenceHighGrayLevelEmphasis	0.6104
	wavelet-HHL-glrlm-RunPercentage	-0.1379
	original-firstorder-RobustMeanAbsoluteDeviation	0.2757

© 2020 Yu Y et al. *JAMA Network Open.*

	wavelet-LHH-glrlm-LongRunLowGrayLevelEmphasis	0.1135
	wavelet-HHL-glrlm-LongRunLowGrayLevelEmphasis	0.3070
	wavelet-LLH-glrlm-RunEntropy	-0.0844
	wavelet-LHL-firstorder-RobustMeanAbsoluteDeviation	0.8842
	wavelet-LLH-glrlm-LongRunEmphasis	0.3321
	wavelet-LLH-glcm-Imc2	0.3802
**Model**	**Intercept/Feature Name**	**Coefficient**
	original-glrlm-RunVariance	-0.0206
	wavelet-LLL-glszm-LargeAreaEmphasis	0.0775
	wavelet-HLL-firstorder-Entropy	0.2048

© 2020 Yu Y et al. *JAMA Network Open.*

	original-glrlm-LongRunEmphasis	0.2054
	wavelet-LLL-glcm-MCC	0.1532
	wavelet-LLH-firstorder-RootMeanSquared	-0.1024
	original-shape-MajorAxisLength	-0.7563
	wavelet-LLH-firstorder-MeanAbsoluteDeviation	0.1581
	wavelet-LHL-glcm-InverseVariance	0.5645
	wavelet-LHL-ngtdm-Coarseness	506.9233
**DWI-ADC sequence signature**	Intercept=-0.0766	β

© 2020 Yu Y et al. *JAMA Network Open.*

Model	Intercept/Feature Name	Coefficie<br>nt
	wavelet-HHH-glrlm-RunPercentage	-0.5917
	diagnostics-Mask-interpolated-VoxelNum	0.2623
	wavelet-LLH-glcm-InverseVariance	0.1956
	wavelet-HLL-glcm-MCC	-0.0220
	wavelet-LLL-firstorder-Energy	0.3723
	wavelet-HLL-firstorder-RobustMeanAbsoluteDeviation	0.1601
	diagnostics-Mask-interpolated-VoxelNum	0.2623
	wavelet-LLH-glcm-InverseVariance	0.1956
	wavelet-HLL-glcm-MCC	-0.0220

© 2020 Yu Y et al. *JAMA Network Open.*

	wavelet-LLL-firstorder-Energy	0.3723
	wavelet-HLL-firstorder-RobustMeanAbsoluteDeviation	0.1601
	wavelet-HHL-glcm-InverseVariance	0.2763
	wavelet-HLH-glszm-LargeAreaEmphasis	0.7292
**Model**	**Intercept/Feature Name**	**Coefficient**
	wavelet-HLH-glcm-InverseVariance	0.2603
	wavelet-HHH-glcm-InverseVariance	-0.0881
	wavelet-LHH-glszm-SmallAreaEmphasis	0.4704
	wavelet-LHH-glrlm-RunEntropy	0.0861
	wavelet-HLL-glszm-ZonePercentage	1.4259

© 2020 Yu Y et al. *JAMA Network Open.*

	original-shape-MajorAxisLength	-0.3813
	wavelet-LLL-glszm-LargeAreaHighGrayLevelEmphasis	-0.0339
	wavelet-LLH-glcm-Imc2	0.1922
	wavelet-HLL-gldm-LargeDependenceLowGrayLevelEmphasis	0.5429
	original-shape-Maximum3DDiameter	-0.9503
	wavelet-HLL-ngtdm-Busyness	-0.7014
	wavelet-LHL-glszm-LargeAreaHighGrayLevelEmphasis	0.7596
	original-ngtdm-Coarseness	1.2341
**Model**	**Intercept/Feature Name**	**Coefficient**

© 2020 Yu Y et al. *JAMA Network Open.*

**Radiomic signature**	Intercept=-3.8489	β
	T1+C sequence signature	4.7965
	T2WI sequence signature	0.4580
	DWI-ADC sequence signature	2.8023
**Clinical signature**	Intercept=-2.9171	β
	Clinical T stage	0.4001
	Clinical N stage	1.8991
	Histological grade	0.1638
	Age	-0.0160

© 2020 Yu Y et al. *JAMA Network Open.*

	HER2 status	-0.1295
**Clinical-radiomic Nomogram**	Intercept=-4.1190	β
**Model**	**Intercept/Feature Name**	**Coefficient**
	Radiomic signature	4.5678
	Clinical signature	4.1789

Abbreviation: ALN, axillary lymph node; T1+C, contrast-enhanced T1-weighted imaging; T2WI, T2-weighted imaging; DWI-ADC, diffusion-weighted imaging quantitatively measured apparent diffusion coefficients; HER2, human epidermal growth factor receptors 2.

© 2020 Yu Y et al. *JAMA Network Open.*

**eTable 6.** Univariable Analysis of Axillary Lymph Node Status in Relation to Clinicopathologic Characteristics									
	**Development cohort**			**Validation cohort**			**Entire cohort**		
	**Axillary Lymph Node Status**			**Axillary Lymph Node Status**			**Axillary Lymph Node Status**		
**Characteristic**	**No. (%) Negative**	**No. (%) Positive**	***P* value**	**No. (%) Negative**	**No. (%) Positive**	***P* value**	**No. (%) Negative**	**No. (%) Positive**	***P* value**
Age, years			.007			.023			< .001
<40	66 (14.1)	81 (21.2)		35(17.2)	44 (27.2)		101 (15.1)	125 (23.0)	
⩾40	401 (85.9)	301 (78.8)		168 (82.8)	118 (72.8)		569 (84.9)	419 (77.0)	
Number of tumors			.885			.027			.189
1	400 (85.7)	325 (85.3)		184 (90.6)	134 (82.7)		584 (87.2)	459 (84.5)	
>1	67 (14.3)	56 (14.7)		19 (9.4)	28 (17.3)		86 (12.8)	84 (15.5)	
Histological type			.123			.389			.385
Invasive ductal carcinoma	410 (87.8)	348 (91.1)		187 (92.1)	145(89.5)		597 (89.1)	493 (90.6)	
Others	57 (12.2)	34 (8.9)		16 (7.9)	17 (10.5)		73 (10.9)	51 (9.4)	
Histological grade			< .001			.576			< .001
Grade 1 (low)	14 (3.7)	4 (1.2)		12 (6.5)	3 (2.2)		26 (4.6)	7 (1.5)	
Grade 2 (intermediate)	211 (55.5)	154 (46.5)		92 (50.0)	76 (55.1)		303 (53.7)	230 (49.0)	

© 2020 Yu Y et al. *JAMA Network Open.*

Grade 3 (high)	155 (40.8)	173 (52.3)		80 (43.5)	59 (42.8)		235 (41.7)	232 (49.5)	
Clinical T stage			< .001			< .001			< .001
T1	205 (44.0)	97 (25.4)		98 (48.3)	44 (27.2)		303 (45.3)	141 (25.9)	
T2	235 (50.4)	244 (63.9)		94 (46.3)	104 (64.2)		329 (49.2)	348 (64.0)	
T3	19 (4.1)	32 (8.4)		9 (4.4)	9 (5.6)		28 (4.2)	41 (7.5)	
T4	7 (1.5)	9 (2.4)		2 (1.0)	5 (3.1)		9 (1.3)	14 (2.6)	
Clinical N stage			< .001			< .001			< .001
N0	391 (83.7)	160 (41.9)		158 (77.8)	66 (40.7)		549 (81.9)	226 (41.5)	
N1	74 (15.8)	185 (48.4)		44 (21.7)	85 (52.5)		118 (17.6)	270 (49.6)	
N2	2 (0.4)	33 (8.6)		0 (0.0)	9 (5.6)		2 (0.3)	42 (7.7)	
N3	0 (0.0)	4 (1.0)		1 (0.5)	2 (1.2)		1 (0.1)	6 (1.1)	
Clinical TNM stage			< .001			< .001			< .001
I	180 (38.6)	52 (13.6)		85 (41.9)	26 (16.0)		265 (39.6)	78 (14.3)	
II	270 (57.9)	268 (70.2)		112 (55.2)	115 (71.0)		382 (57.1)	383 (70.4)	
III	16 (3.4)	62 (16.2)		6 (3.0)	21 (13.0)		22 (3.3)	83 (15.3)	
ER status			.341			.632			.601
Negative	68 (14.6)	65 (17.0)		35 (17.4)	25 (15.5)		103 (15.5)	90 (16.6)	

© 2020 Yu Y et al. *JAMA Network Open.*

Positive	397 (85.4)	317 (83.0)		166 (82.6)	136 (84.5)		563 (84.5)	453 (83.4)	
PR status			.935			.754			.911
Negative	130 (28.0)	108 (28.3)		63 (31.3)	48 (29.8)		193 (29.0)	156 (28.7)	
Positive	334 (72.0)	274 (71.7)		138 (68.7)	113 (70.2)		472 (71.0)	387 (71.3)	
HER2 status			.337			< .001			< .001
Negative	296 (71.5)	228(68.3)		128 (72.3)	78 (54.5)		424 (71.7)	306 (64.2)	
Positive	118 (28.5)	106 (31.7)		49 (27.7)	65 (45.5)		167 (28.3)	171 (35.8)	
Ki67 status			.350			.867			.488
<15	125 (27.0)	87 (22.9)		54 (27.0)	36 (22.4)		179 (27.0)	123 (22.7)	
15-35	202 (43.6)	169 (44.5)		86 (43.0)	79 (49.1)		288 (43.4)	248 (45.8)	
35-55	61 (13.2)	66 (17.4)		24 (12.0)	25 (15.5)		85 (12.8)	91 (16.8)	
>55	75 (16.2)	58 (15.3)		36 (18.0)	21 (13.0)		111 (16.7)	79 (14.6)	
Molecular subtypes			.102			.489			.326
Luminal A	95 (21.2)	59 (16.1)		39 (20.1)	21 (13.5)		134 (20.9)	80 (15.3)	
Luminal B	291 (65.0)	248 (67.8)		121 (62.4)	114 (73.1)		412 (64.2)	362 (69.3)	
HER2-positive	33 (7.4)	31 (8.5)		12 (6.2)	16 (10.3)		45 (7.0)	47 (9.0)	
Triple negative	29 (6.5)	28 (7.7)		22 (11.3)	5 (3.2)		51 (7.9)	33 (6.3)	

© 2020 Yu Y et al. *JAMA Network Open.*

Abbreviations: TNM, tumor–node–metastasis; HER2, human epidermal growth factor receptors 2; ER, estrogen receptor; PR, progesterone receptors; Ki67, proliferation marker protein Ki-67.

© 2020 Yu Y et al. *JAMA Network Open.*

eTable 7. Multivariate Analysis of Axillary Lymph Node Metastasis in Relation to Clinical Signature and Radiomic Signature in the Development Cohort

**Signature**	**Multivariate Analysis**	
	**OR (95%CI)**	***P* value**
Radiomic signature (high-risk/low-risk)	6.88 (3.90-12.33)	< .001
Clinical signature (high-risk/low-risk)	9.82 (5.22-19.29)	< .001

Abbreviations: OR, Odds ratio; CI, confidence interval.

© 2020 Yu Y et al. *JAMA Network Open.*

eTable 8. The Performance of Each Signature Constructed With Different Algorithm for DFS Prediction

		**Feature Selection**		**LASSO**			**Random Forest**	
**Signature**	**Signature Performance**	**Signature Building**		**Cox Regression**			**Cox Regression**	
		**Cohort**		**Development Cohort**	**Validation Cohort**		**Development Cohort**	**Validation Cohort**
T1+C sequence radiomic signature								
	1-year AUC			0.84	0.44		0.81	0.58
	2-year AUC			0.80	0.50		0.78	0.60
	3-year AUC			0.75	0.53		0.73	0.63
T2WI sequence radiomic signature								
	1-year AUC			0.74	0.61		0.79	0.53
	2-year AUC			0.70	0.62		0.79	0.53
	3-year AUC			0.64	0.53		0.76	0.47
DWI-ADC sequence radiomic signature								
	1-year AUC			0.69	0.73		0.75	0.70
	2-year AUC			0.67	0.77		0.78	0.72
	3-year AUC			0.61	0.82		0.77	0.70
Radiomic signature								
	1-year AUC			0.83	0.54		0.80	0.68

© 2020 Yu Y et al. *JAMA Network Open.*

	2-year AUC			0.82	0.64		0.83	0.74
	3-year AUC			0.78	0.66		0.81	0.73

Abbreviations: LASSO, Least absolute shrinkage and selection operator; AUC, area under the receiver operating characteristics curve; T1+C, contrast-enhanced T1-weighted imaging; T2WI, T2-weighted imaging; DWI-ADC, diffusion-weighted imaging quantitatively measured apparent diffusion coefficients.

© 2020 Yu Y et al. *JAMA Network Open*.

eTable 9. Essential Radiomic Features and Formula Composition for DFS Prediction

Model	Feature Name	Coefficient
**T1+C sequence signature**		
	wavelet-HLL-glszm-SizeZoneNonUniformity	0.8513
	wavelet-HHH-glrlm-RunPercentage	-0.0566
	wavelet-LHH-firstorder-Uniformity	-0.0849
	wavelet-HHL-glszm-ZoneEntropy	-1.1890
	wavelet-LHH-glszm-SizeZoneNonUniformity	0.8160
	wavelet-LHL-glrlm-RunLengthNonUniformity	0.0644

© 2020 Yu Y et al. *JAMA Network Open.*

	wavelet-HHH-glszm-SizeZoneNonUniformity	0.4476
	wavelet-HLH-firstorder-Kurtosis	0.1274
	wavelet-HHL-glszm-GrayLevelNonUniformity	-2.1320
**Model**	**Feature Name**	**Coefficient**
	wavelet-LHH-glrlm-GrayLevelNonUniformity	0.2053
	wavelet-HHL-glszm-SizeZoneNonUniformity	47.7500
	wavelet-LHH-firstorder-Kurtosis	0.2448
	wavelet-HHL-glszm-SizeZoneNonUniformityNormalized	-44.0300
	wavelet-LLH-glszm-SmallAreaLowGrayLevelEmphasis	0.5448
	original-shape-Maximum2DDiameterColumn	0.5991

© 2020 Yu Y et al. *JAMA Network Open.*

	wavelet-LLH-glcm-Contrast	-0.1834
	wavelet-LHH-ngtdm-Strength	-0.0083
	wavelet-HLH-glszm-SizeZoneNonUniformity	-1.0020
	wavelet-LHH-firstorder-10Percentile	-0.3406
	original-shape-MeshVolume	0.2630
	original-shape-Sphericity	0.2054
	wavelet-LLL-firstorder-Energy	-1.7020
	wavelet-LLH-firstorder-TotalEnergy	-0.4103
**Model**	**Feature Name**	**Coefficient**
	wavelet-HLH-firstorder-10Percentile	0.3420
	wavelet-HHL-ngtdm-Contrast	0.1668

© 2020 Yu Y et al. *JAMA Network Open.*

	wavelet-LLH-glszm-ZoneEntropy	-0.0813
	wavelet-HHH-glszm-ZoneEntropy	-0.7329
	wavelet-LLH-firstorder-InterquartileRange	-1.1560
	wavelet-HLH-firstorder-Uniformity	-0.6731
	wavelet-HHH-firstorder-Kurtosis	0.5630
**T2WI sequence signature**		
	wavelet-HLL-glszm-GrayLevelNonUniformity	-0.0563
	wavelet-LHH-glrlm-GrayLevelNonUniformity	-0.6585
	wavelet-HHL-glszm-SizeZoneNonUniformity	3.7646
	wavelet-HHH-glszm-SizeZoneNonUniformityNormalized	-2.0030

© 2020 Yu Y et al. *JAMA Network Open.*

	wavelet-HLL-firstorder-Mean	-4.9581
**Model**	**Feature Name**	**Coefficient**
	original-shape-Sphericity	0.0977
	wavelet-HLL-firstorder-Uniformity	1.1684
	wavelet-LLH-glszm-SizeZoneNonUniformity	1.1128
	wavelet-HLL-firstorder-Kurtosis	-0.2916
	wavelet-HHH-glszm-ZoneEntropy	1.8131
	wavelet-LHL-firstorder-TotalEnergy	4.1148
	original-shape-Maximum2DDiameterColumn	3.9735
	wavelet-HHL-glrlm-LongRunHighGrayLevelEmphasis	0.4227
	wavelet-HHH-glrlm-RunLengthNonUniformity	-1.9274

© 2020 Yu Y et al. *JAMA Network Open.*

	original-shape-MeshVolume	0.1625
	wavelet-HHL-glszm-ZoneEntropy	0.9950
	wavelet-HLH-glrlm-RunLengthNonUniformity	2.7230
	wavelet-HHL-glrlm-GrayLevelNonUniformity	0.7743
	wavelet-LLH-glszm-ZoneEntropy	0.7662
**Model**	**Feature Name**	**Coefficient**
	wavelet-HHH-glrlm-RunPercentage	-0.0494
	wavelet-HHH-glrlm-GrayLevelNonUniformity	0.6484
	wavelet-HLH-glszm-SizeZoneNonUniformity	-0.8657
	wavelet-HHL-glszm-GrayLevelNonUniformity	-0.2980
	wavelet-HLH-glrlm-GrayLevelVariance	0.6176

© 2020 Yu Y et al. *JAMA Network Open.*

	wavelet-HLH-firstorder-Kurtosis	-0.0233
	wavelet-LLH-firstorder-TotalEnergy	-3.7440
	wavelet-HLH-glrlm-GrayLevelNonUniformity	-0.1860
	wavelet-HLH-firstorder-Uniformity	-1.1006
	wavelet-LHH-firstorder-Kurtosis	-0.8432
	wavelet-HLH-glszm-ZoneEntropy	-5.0645
**DWI-ADC sequence signature**		
**Model**	**Feature Name**	**Coefficient**
	wavelet-LHL-glrlm-RunLengthNonUniformityNormalized	-0.1017

© 2020 Yu Y et al. *JAMA Network Open.*

	wavelet-HHL-glszm-GrayLevelNonUniformity	-0.6127
	wavelet-HHL-gldm-DependenceNonUniformity	-0.0277
	wavelet-LLH-glcm-JointEntropy	-2.2394
	wavelet-HHH-glrlm-RunLengthNonUniformity	1.8230
	wavelet-HHH-glszm-SizeZoneNonUniformity	-3.0838
	wavelet-LHL-glszm-ZoneEntropy	1.6147
	wavelet-LHH-glrlm-RunLengthNonUniformity	-0.1816
	original-firstorder-Mean	0.2905
	wavelet-HLL-firstorder-TotalEnergy	1.1169
	wavelet-LHH-ngtdm-Contrast	0.1275
	wavelet-HLL-firstorder-Mean	0.1465

© 2020 Yu Y et al. *JAMA Network Open.*

	wavelet-HLH-glszm-SizeZoneNonUniformity	1.3711
	wavelet-HLH-firstorder-Kurtosis	-0.0953
**Model**	**Feature Name**	**Coefficient**
	wavelet-LLH-glrlm-GrayLevelNonUniformityNormalized	-0.2268
	wavelet-LHL-firstorder-Uniformity	-0.4446
	wavelet-HHL-ngtdm-Contrast	0.0941
	wavelet-HLL-glrlm-RunLengthNonUniformity	-0.4508
	wavelet-HHL-firstorder-TotalEnergy	-1.8425
	wavelet-HLH-glszm-LargeAreaLowGrayLevelEmphasis	0.2196
	diagnostics-Mask-interpolated-BoundingBox	-0.7884

© 2020 Yu Y et al. *JAMA Network Open.*

	wavelet-HHL-glszm-ZoneEntropy	3.7485
	wavelet-LLH-firstorder-Kurtosis	0.1644
	wavelet-HHL-glszm-LargeAreaLowGrayLevelEmphasis	0.4181
	wavelet-HLL-glszm-ZoneEntropy	-2.7319
	wavelet-HLH-glszm-GrayLevelNonUniformity	-0.0254
	wavelet-LHH-gldm-LargeDependenceEmphasis	0.1046
	wavelet-LLH-firstorder-Uniformity	-1.3138
**Model**	**Feature Name**	**Coefficient**
	original-shape-MinorAxisLength	0.4667
	original-shape-Sphericity	0.4792

© 2020 Yu Y et al. *JAMA Network Open.*

**Radiomic signature**		
	T1+C sequence signature	0.5075
	T2WI sequence signature	0.2717
	DWI-ADC sequence signature	0.6309
**Clinical signature**		
	Number of tumors	0.9420
	Histological grade	0.3346
	Pathological T stage	-0.0160
	Pathological N stage	0.8280
	PR status	-0.4022

© 2020 Yu Y et al. *JAMA Network Open.*

	Ki-67 expression level	0.3440
**Model**	**Feature Name**	**Coefficient**
	Type of surgery	-0.5657
**Clinical-radiomic Nomogram**		
	Radiomic signature	0.7085
	Clinical signature	0.6444
Abbreviation: DFS, disease-free survival; T1+C, contrast-enhanced T1-weighted imaging; T2WI, T2-weighted imaging; DWI-ADC, diffusion-weighted imaging quantitatively measured apparent diffusion coefficients; PR, progesterone receptors; Ki-67, proliferation marker protein Ki-67.		

© 2020 Yu Y et al. *JAMA Network Open.*

eTable 10. Multivariate Analysis of Clinical Signature and Radiomic Signature With DFS in the Development Cohort

Signature	Multivariate Analysis	
	**HR (95%CI)**	***P* value**
Radiomic signature (low-risk/high-risk)	0.16 (0.07-0.36)	< .001
Clinical signature (low-risk/high-risk)	0.16 (0.07-0.40)	< .001

Abbreviations: DFS, disease-free survival; HR, Hazard ratio; CI, confidence interval.

© 2020 Yu Y et al. *JAMA Network Open.*
